# LDHA-mediated glycolysis in stria vascularis endothelial cells regulates macrophages function through CX3CL1-CX3CR1 pathway in noise-induced oxidative stress

**DOI:** 10.1038/s41419-025-07394-6

**Published:** 2025-02-03

**Authors:** Ying Yi, Min-Yu Wu, Kai-Tian Chen, An-Hai Chen, Lin-Qiu Li, Qin Xiong, Xian-Ren Wang, Wen-Bin Lei, Guan-Xia Xiong, Shu-Bin Fang

**Affiliations:** Otorhinolaryngology Hospital, The First Affiliated Hospital, Sun Yat-sen University, 58 Zhongshan Road II, Guangzhou, Guangdong 510080 China

**Keywords:** Mechanisms of disease, Metabolism

## Abstract

According to the World Health Organization, more than 12% of the world’s population suffers from noise-induced hearing loss (NIHL). Oxidative stress-mediated damage to the stria vascularis (SV) is one of the pathogenic mechanisms of NIHL. Recent studies indicate that glycolysis plays a critical role in endothelial cells (ECs)-related diseases. However, the specific role of glycolysis in dysfunction of SV-ECs remain largely unknown. In this study, we investigated the effects of glycolysis on SV-ECs in vitro and on the SV in vivo. Our previous research identified the glycolysis pathway as a potential mechanism underlying the SV-ECs injuries induced by oxidative stress. We further examined the expression levels of glycolytic genes in SV-ECs under H_2_O_2_ stimulation and in noise-exposed mice. We found that the gene and protein expression levels of glycolytic-related enzyme LDHA significantly decreased at early phase after oxidative stress injury both in vitro and in vivo, and exhibited anti-inflammatory effects on macrophages (Mφ). Moreover, we analyzed the differential secretomes of SV-ECs with and without inhibition of LDHA using LC-MS/MS technology, identifying CX3CL1 as a candidate mediator for cellular communication between SV-ECs and Mφ. We found that CX3CL1 secretion from SV-ECs was decreased following LDHA inhibition and exhibited anti-inflammatory effects on Mφ via the CX3CR1 pathway. Similarly, the pro-inflammatory effect of LDHA-overexpressing SV-ECs was attenuated following inhibition of CX3CL1. In conclusion, our study revealed that glycolysis-related LDHA was reduced in oxidative stress-induced SV-ECs, and that LDHA inhibition in SV-ECs elicited anti-inflammatory effects on Mφ, at least partially through the CX3CL1-CX3CR1 pathway. These findings suggest that LDHA represent a novel therapeutic strategy for the treatment of NIHL.

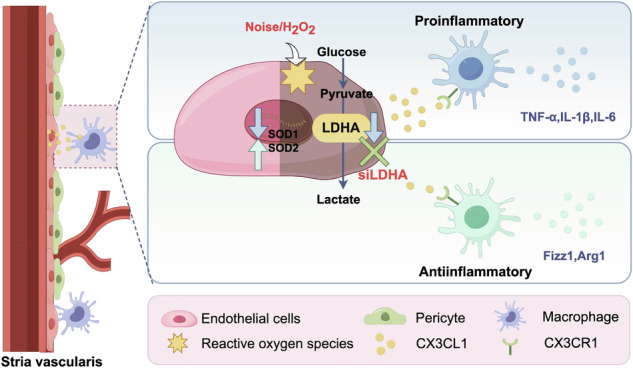

## Introduction

Noise-induced hearing loss (NIHL) is characterized by an elevation of hearing thresholds, permanent loss of cochlear hair cells in the inner ear, auditory processing disorders, and non-auditory symptoms such as sleep and cognitive disorders [1]. The World Health Organization reports that an estimated 1.1 billion young people are at risk of permanent hearing loss due to prolonged exposure to loud music. This issue seriously diminishes the quality of life for NIHL individuals and imposes substantial economic burden on society [2, 3]. However, there are currently no FDA-approved medications for the treatment of NIHL, and the mechanisms underlying its pathogenesis remain to be further elucidated [4].

It is widely recognized that oxidative stress-induced damage to the cochlea is one of the key pathogenic mechanisms of NIHL, with extensive research focusing on inner ear hair cells [5]. The stria vascularis (SV), located on the lateral wall of the cochlea, plays a crucial role in maintaining the endocochlear potential and ion balance of the cochlea [6]. Prior studies have shown that acoustic trauma also causes oxidative-related damages of SV, particularly affecting the blood-labyrinth barrier (BBB), and subsequently leading to hearing loss [6–8]. Endothelial cells (ECs) in the SV (SV-ECs) are vital components of the BBB and are susceptible to noise-induced oxidative stress, which leads to the breakdown of SV-ECs intercellular connections and disrupts inner ear homeostasis [6, 9]. However, the mechanisms underlying oxidative-induced injuries in SV-ECs and their interactions with other inner ear cells remain largely unexplored.

The cellular response to oxidative stress is an energy-consuming process [10]. Physiologically, 85% of the energy for SV-ECs is derived from glycolysis, which metabolizes glucose into lactic acid via lactate dehydrogenase A (LDHA) and supports essential functions such as molecular transmission, connection maintenance, and overall normal function of organs [11]. It has been reported that ATP levels decrease in cochlear tissue after noise exposure, correlating with permanent loss of cochlear hair cells [12, 13]. Our previous study revealed that the glycolysis pathway is one of the pathways involved in the secretomes of SV-ECs following oxidative stress [14]. Also, recent research demonstrated that reduced glycolysis-related NADPH levels are strongly associated with outer hair cell loss due to accumulation of reactive oxygen species (ROS) [15]. Additionally, the glycolysis pathway involved in LDHA expression dynamically changes between hair cells and supporting cells during cochlear development [16]. Mice with LDHB knocked out showed earlier and more severe hair cell degeneration, spiral ganglion neuron death, and loss of fibroblasts in the lateral wall [17]. All these data suggested that glycolysis played an important role in pathogenesis of sensorineural hearing loss, especially the LDH, a tetrameric enzyme encoded by the two genes LDHA and LDHB. However, it remains unclear whether SV-ECs utilize glycolysis such as LDHA to engage in intracellular crosstalk with other cells within microenvironment of NIHL.

Macrophages (Mφ) are immune cells widely distributed across various organs and tissues in the human body, playing a crucial role in the onset and progression of diseases [18]. Under normal circumstances, resident Mφ, predominantly originating from yolk sac progenitor cells or hematopoietic stem cells during embryonic development, are the primary cell types within the cochlea [19–21]. They are distributed among the sensory, neuronal, and supporting tissues in the cochlea, playing a critical role in maintaining the homeostasis of the inner ear environment. However, in the context of NIHL, the number of Mφ in the cochlea significantly increases 3 days after noise-induced injury, peaking approximately 2 weeks after the noise damage. These Mφ are mainly recruited to the cochlear tissue from circulating monocytes in the blood, and they undergo significant morphological changes from amoeboid to branched forms [19, 22]. Alterations in the number and function of noise-induced inner ear Mφ can lead to a persistent inflammatory response, causing ongoing damage to inner ear hair cells and neurons, thereby exacerbating hearing loss [23–26]. However, the immunoregulatory mechanisms underlying the dysfunction of inner ear Mφ during NIHL remain to be further investigated. It has been widely reported that vascular ECs located in organs such as the cardiovascular [27] and pulmonary [28] system were able to exhibit interactions with Mφ through specific factors. Thereby, we speculated that oxidative stress-mediated EC injury was engaged in intercellular crosstalk with inner ear Mφ.

In this study, we sought to clarify the role of glycolysis-related LDHA in oxidative stress-induced SV-ECs injuries, and explore their crosstalk with inner ear Mφ in the setting of NIHL.

## Methods

### Development of NIHL mouse model

C57BL/6 male mice (3–5w) were purchased from The Laboratory Animal Center, Sun Yat-sen University (Guangdong, Guangzhou, China). The mice were randomly divided into two groups and housed in separate cages. One group was exposed to a broadband noise (2–20 kHz) at a sound pressure level (SPL) of 120 dB for 2 h in a ventilated sound insulation cabinet. Briefly, unrestrained mice were placed within a sound chamber equipped with a loudspeaker (JBL, Los Angeles, CA, USA) driven by a power amplifier (Yamaha, Tokyo, Japan). Sound levels within the chamber were continuously monitored with a sound level meter throughout the noise exposure period. Control mice were placed in the same chamber without noise exposure for 2 h. Samples sizes of the mice in our study were estimated from our previous studying experiences, and no blinding was conducted.

### Culture of SV-ECs, RNA interference and lentivirus construction

The detailed culturing procedures of SV-ECs were described in our previous study [14], which was modified as previously reported [29]. Briefly, we established an oxidative stress model of SV-ECs by treating the cells with 500 μM H_2_O_2_. For RNA interference, SV-ECs were transfected with 50 nM siRNA (siLDHA/siCX3CL1/siNC) using the riboFECT CP Transfection Kit (RiboBio, Guangzhou, Guangdong, China) according to manufacturer’s instructions. The lentivirus for LDHA (LV-LDHA) overexpression construction (lentiviral vector GV492: Ubi-MCS-3FLAG-CBh-gcGFP-IRES-puromycin) was produced by Genechem Company (Shanghai, China). SV-ECs were infected with LV-LDHA with a multiplicity of infection (MOI) of 50 at 50% confluence. After 72 h, the expression of enhanced green fluorescent protein (GFP) was observed by fluorescence microscope at a ×100 magnification (Leica). Subsequently, a concentration of 1 μg/mL puromycin was used to select the SV-ECs that successfully expressed LDHA.

### Intracellular reactive oxygen species assay

Upon reaching 80% confluence, SV-ECs cultured in 24-well plates were exposed to 500 µM H_2_O_2_ or left untreated. Adherent cells were washed with phosphate buffered saline (PBS, Gibco, Carlsbad, CA, USA) after 2 h, and fresh media containing 10 μM 2′,7′-dichlorodihydrofluorescein diacetate (DCFH-DA) probes (Sigma‒Aldrich, St. Louis, MO, USA) was added and incubated for 30 min at 37 °C in a humidified incubator with 10% CO_2_. Subsequently, the cells were washed with ice-cold PBS and protected from light. The fluorescence of DCFH-DA was detected by BioTek Gene microplate reader (BioTek, Winooski, VT, USA).

### Cell viability assay

Cells were seeded at a density of 1 ×10^4^ per well in 96-well plates and cultured with or without LDHA inhibitor az-33(Abcam, Cambridge, UK) or siRNA-LDHA. Cell viability was accessed using the Cell Counting Kit-8 (Yeasen, Shanghai, China) colorimetric assay according to the manufacturer’s protocol. Absorbance was measured at 450 nm using a BioTek Gene microplate reader (BioTek).

### Transepithelial electrical resistance (TEER)

A total of 1 ×10^5^ cells were seeded into the upper chambers of transwell 24-well plates equipped with 0.4 μm pore filters (Corning, Corning, NY, USA). The apical compartments received 0.2 mL of medium, while the basal compartments were filled with 0.6 mL. The cells were incubated until steady-state transepithelial electrical resistance (TEER) was achieved. Subsequently, the filters replaced with fresh Hank’s Balanced Salt Solution (HBSS, Carlsbad, CA, USA) with or without 500 μM H_2_O_2_ for 2 h, followed by treatment with az-33 or siLDHA for 72 h at 37 °C. TEER measurements were obtained using an EVOM_3_ meter (WPI, Sarasota, FL, USA).

### Migration assay

The SV-ECs were seeded at a density of 5 ×10^4^ cells/well into the upper chamber of transwell 24-well plates (Corning) fitted with 8 μm pore filters and cultured with low serum medium (containing 1% Fetal Bovine Serum). The cells were subjected to treatments as described in the TEER assay. The lower chamber was filled with a complete medium (containing 10% Fetal Bovine Serum). After 12 h of incubation, cells adhering to the upper surface of the filter membranes were removed, and cells that had migrated to the lower surface were stained with 0.5% crystal violet for 15 min. The extent of cell migration was then examined using an optical microscope (Leica, Weizler, Hesse, Germany).

### Tube formation assay

A total of 1 ×10^5^ SV-ECs were seeded into 96-well plates pre-coated with 60 μL of growth factor-reduced Matrigel (BD Biosciences, Franklin Lake, NJ, USA). The plates were then subjected to various treatments, as described in the TEER assay, to assess the formation of tube-like structures. Tube formation was observed using an inverted microscope (Leica). The total number of branching points and the overall tube length were quantified by ImageJ software.

### Enzyme linked immunosorbent assay (ELISA)

The levels of TNF-α, IL-6, and IL-1β in the supernatants were quantified using ELISA kits (NeoBioscience, Shenzhen, Guangdong, China) according to the manufacturer’s instructions. Absorbance at 450 nm was measured using a BioTek Gene microplate reader (BioTek).

### Lactate release assay

SV-ECs were seeded at a density of 2 ×10^4^ cells per well in 24-well plates. Upon reaching 60% confluence, the medium was replaced with 1 mL of fresh complete medium per well, followed by treatments as described in the TEER assay. Supernatants were collected for lactate measurement using a lactate assay kit (KeyGEN BioTECH, Nanjing, Jiangsu, China).

### Auditory brainstem response measurement (ABR)

All mice were anesthetized with an intraperitoneal injection of ketamine (100 mg/kg) and xylazine (10 mg/kg), and ABR measurements were performed by inserting subdermal needle electrodes. Acoustic signals were generated, and the responses were processed using the Tucker-Davis Technologies (TDT System III, Alachua, FL USA). ABR recordings were performed at click frequencies of 8, 16, 24 and 32 kHz. The minimum stimulation intensity required to elicit Wave II was determined as the ABR threshold.

### Intratympanic injection

C57BL/6 mice aged 3–5 weeks were anesthetized with an intraperitoneal injection of a mixture of ketamine (100 mg/kg) and xylazine (10 mg/kg). The left surgical area was sterilized with iodophor. Under a microscope, an incision was made 1-2 mm posterior to the left ear to expose the skin and subcutaneous tissue. The great auricular nerve and sternocleidomastoid muscle were identified and separated. The posterior wall of the acoustic bulla was exposed and drilled through using a 1 mL syringe needle. Finally, an injection was administered via the round window.

### Tissue preparations

Following ABR recordings, the deeply anesthetized mice were decapitated, and their cochleae were excised and fixed by immersion in 4% paraformaldehyde overnight at 4 °C. The cochleae were then decalcified in 4% sodium ethylenediaminetetraacetic acid (EDTA, Sigma‒Aldrich) for 4 days, followed by sequential incubation in 5%, 10%, and 20% sucrose solutions. Some cochleae were embedded in O.C.T. compound (Sakura, Tokyo, Japan), cryosectioned at a thickness of 7 μm, and stored at −20 °C for hematoxylin and eosin (HE) staining or immunofluorescence analysis. Other cochleae were directly processed for surface preparations. For RNA and protein extraction, cochlear tissues were dissected and stored at −80 °C.

### Measurement of stria vascularis thickness

Stria vascularis thickness measurement was obtained from cochlear sections with H&E staining (Solarbio, Wuhan, Hubei, China). Measurements were performed on three images containing the middle turn of the cochlea for each mouse, and the data were aggregated to calculate the mean thickness.

### Immunofluorescence

Tissues were fixed for 30 min at room temperature in 4% paraformaldehyde (Sigma‒Aldrich) in PBS, and then incubated with 0.3% Triton X-100 and 10% goat-serum in PBS for 30 min for permeabilization and blocking, respectively. Subsequently, tissue was incubated with primary antibody against CX3CL1(AF537-SP R&D Systems, Minneapolis, MN, USA), IBa1(ab178846, Abcam, Cambridge, UK), 3NT (bs-8551R, Bioss, Beijing, China), CD86 (13395-1-AP, Proteintech, Wuhan, Hubei, China), and CD31 (28083-1-AP, Proteintech) overnight, and incubated with secondary antibodies or FITC-BSIB4 (L2895, Thermo Fisher Scientific, San Jose, CA, USA) for 1 h at room temperature. Nuclei were stained with 4´,6-diamidino-2-phenylindole (DAPI; Roche, Switzerland). Imaging was conducted with a DM4B microscope (Leica).

### Western blot

Cochlear tissues and cultured cells were homogenized on ice-cold radioimmunoprecipitation assay lysis buffer for 30 min, then centrifuged at 14,000 × *g* at 4 °C for 15 min, and the supernatants were collected. Protein concentrations were determined according to BCA assay instruction (Beyotime, Shanghai, China). Protein was separated via SDS–polyacrylamide gel electrophoresis and transferred to PVDF membranes (Millipore, Boston, MA, USA), which were blocked with 5% skim milk. The membranes were then incubated overnight with the following primary antibodies as indicated: GPI (27978, 1:1000, SAB, Greenbelt, MD, USA), ALDOA (ab252953, 1:1000, Abcam), TPI1 (54087, 1:1000, SAB), G3P (1:1000, Abcam), PGAM1 (49976, 1:1000, SAB), PKM (7067T, 1:1000, CST, Danvers, MA, USA), LDHA (19987-1-AP, 1:1000, Abcam), PGK1 (ab199438, 1:1000, Abcam,) CX3CL1(29282, 1:500, SAB) and β-actin (20536-1-AP, 1:2000, Proteintech) overnight. Following three 10-minute washes with TBST, the membranes were incubated with the appropriate secondary antibody (1:2000, Cowin Biotech) for 1 h. Immunoreactive bands were visualized using enhanced chemiluminescence (Millipore). Band intensities were quantified and normalized to β-actin as the loading control using ImageJ software.

### Quantitative reverse transcription polymerase chain reaction (qRT-PCR)

Total RNA was extracted using Trizol (Invitrogen, Carlsbad, CA, USA) following the manufacturer’s protocol. Complementary DNA (cDNA) was synthesized using the Evo M-MLV RT reagent premix Kit (Accurate Biotechnology, Changsha, Hunan, China). Then, qPCR was performed using the SYBR Green Pro Taq HS premixed qPCR Kit (Accurate Biotechnology). Data were calculated using the 2^−ΔΔCt^ method, with mRNA levels were normalized to β-actin. The primers used in the study are listed in Supplementary Table S1.

### Isolation of single cells for flow cytometry analysis

The samples for flow cytometry analysis included control, mice exposed to noise 3 days, with each group comprising 4 cochleae. The procedures for obtaining single cells were adapted from a previous study [30]. Briefly, cochleae were dissected to isolate the lateral wall, modiolus with the spiral ganglion, spiral limbus, inner sulcus, organ of Corti, and outer sulcus. These tissues were placed in a digestion solution (Stemcell, Vancouver, Canada), and incubated at 37 °C for 30 min, with pipetting every 10 min. The cells were then filtered using a 70-μm strainer (BD) and centrifuged at 500 g at 4 °C for 10 min. The resulting single-cell suspensions were used for flow cytometry analysis. For the flow cytometry analysis of CD86 and CD206 in single cells from mouse cochlea, cells were stained with markers F4/80-FITC (Biolegend, San Diego, CA, USA), CD206 -PC7 (Biolegend, San Diego, CA, USA) and CD86-APC (Biolegend, San Diego, CA, USA). Gating strategies for flow cytometry analyses of Mφ in cochleae are shown in Supplementary Fig. S3B.

### Cell supernatants for sequencing and stimulation

The detailed procedures for collecting cell supernatant were described in our previous study [14]. Briefly, the SV-ECs were treated with siRNA or lentivirus under different conditions followed by 24 h of serum-free medium. The supernatant was collected and centrifuged at 2000 × *g* for 5 min to remove cell debris. Subsequently, the supernatants were concentrated by centrifugation at 8000 × *g* for 2 h at 4 °C using a 3 kDa MWCO ultrafiltration tube (Thermo Fisher Scientific). The proteomics of cell supernatants collected from SV-ECs treated with siLDHA and siNC were performed using the high-resolution mass spectrometer, as we described in our previous study [14]. The cell supernatants of LV-LDHA and siCX3CL1 were used to stimulate Mφ.

### Statistical analyses

Statistical analyses were conducted using Prism software (GraphPad Software, La Jolla, CA, USA). All experiments were repeated at least three independent times. Data are presented as mean ± SD for each group. As indicated, statistical significance was determined using an independent two-tailed Student’s *t* test or Mann–Whitney test for single comparisons or one-way ANOVA with Tukey’s correction for multiple comparisons. A *P* value less than 0.05 was considered statistically significant.

## Results

### Function of SV-ECs was disrupted by noise-induced oxidative stress

We first established a mouse model of NIHL characterized by PTS (permanent threshold shift). Significant declines in hearing function were observed in 1, 3, and 14 days post-noise exposure (Supplementary Fig. S1A-B). Additionally, a marked decrease in apoptosis of outer hair cells was noted at 14 days post-exposure (Supplementary Fig. S1C). Concurrently, protein expression of 3-NT, an indicator of oxidative stress, was significantly elevated at 1 day after noise exposure in the cochleae (Supplementary Fig. S1D). These data suggested that oxidative stress is induced in the inner ear following NIHL.

**Fig. 1:**
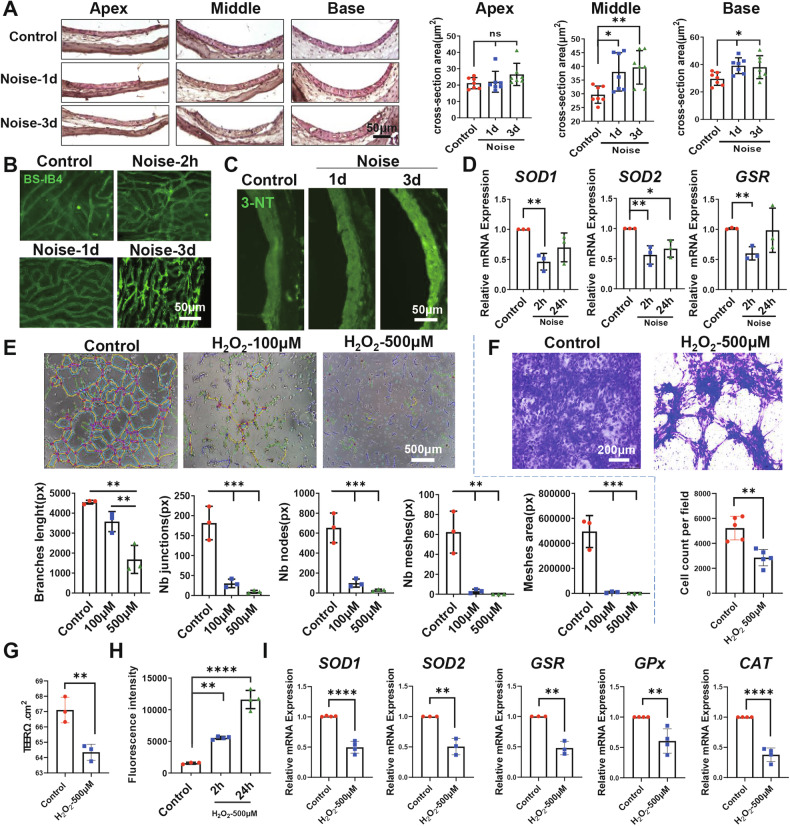
The function of SV-ECs injury occurred in the acute phase following oxidative stress. **A** H&E staining of cochlear lateral walls and quantifications of cross-section area of SV in all cochlear turns by Image J. Significant increases in middle and basal turns of SV after noise exposure were found (*n* = 7) (Scale bar, 50 μm). **B** Representative confocal figures of BS-IB4 (green) indicating stronger fluorescence in damaged SV-ECs in 3 days after noise. (Scale bar, 50μm). **C** Representative confocal pictures of 3-NT (green) showing stronger fluorescence of SV in 3 days post-noise exposure (Scale bar, 50 μm). **D** Relative mRNA levels of anti-oxidative genes, including SOD1, SOD2 and GSR, were decreased in SVs of cochleae after noise exposure (*n* = 3). **E** Angioplasty experiments revealed significant reductions in the branches length, number of junctions, number of nodes, number of meshes and area of endothelial cell angioplasty after stimulation with 100 μM and 500 μM H_2_O_2_ for 2 h (scale bar, 500 μm) (*n* = 3). **F** Representative images and quantification of transwell migration assays of SV-ECs (scale bar, 200 μm) (*n* = 5). **G** Transepithelial electrical resistance (TEER) decreased in SV-ECs treated with 500 μM H_2_O_2_ for 2 h (*n* = 3). **H** ROS fluorescence intensity was increased in SV-ECs stimulated by 500 μM H_2_O_2_ for 2 h and 24 h (*n* = 4). **I** Relative mRNA levels of anti-oxidative, including *SOD1*, *SOD2, GSR, CAT and GPX*, were decreased in SV-ECs at 2 h following treatment with 500 μM H_2_O_2_ (*n* = 3). The data is expressed as Mean ± SD. *P < 0.05, **P < 0.01, *** *P* < 0.001, **** *P* < 0.0001 analyzed by Unpaired *t* test, SV stria vascularis, ECs endothelial cells, ns not significant, SOD1 Superoxide Dismutase 1, SOD2 Superoxide Dismutase 2, GSR Glutathione-disulfide reductase, GPx Glutathione peroxidases, CAT Catalase, qRT-PCR Quantitative Reverse Transcription Polymerase Chain Reaction.

To investigate the impact of noise-induced oxidative stress on SV, we used frozen section HE staining to examine the cochleae and found that the structure of the SV on the lateral wall of the cochleae was disrupted and swollen, with a significant increase in the cross-sectional area of blood vessels at 1 and 3 days post-noise exposure, particularly in the middle and basal turns of the cochlea (Fig. 1A). Notably, destruction of SV-ECs (Fig. 1B) and increased expression of 3-NT (Fig. 1C) were observed at 1 day post-noise exposure. Additionally, a decrease in the mRNA expression levels of antioxidant genes, including SOD1, SOD2, and GSR, was noted following noise exposure (Fig. 1D). To further examine the effects of oxidative stress on SV-ECs function, we established an in vitro model of SV-ECs oxidative stress injury induced by H_2_O_2_. Assessments of tube formation, migration, and barrier integrity of SV-ECs confirmed that oxidative stress mediates functional damage to SV-ECs (Fig.1E–G). Moreover, we observed an increase in ROS levels (Fig. 1H), alongside a decrease in the expression of antioxidant genes in SV-ECs at 2 h after treatment with 500 μM H_2_O_2_ (Fig.1I).

**Fig. 2:**
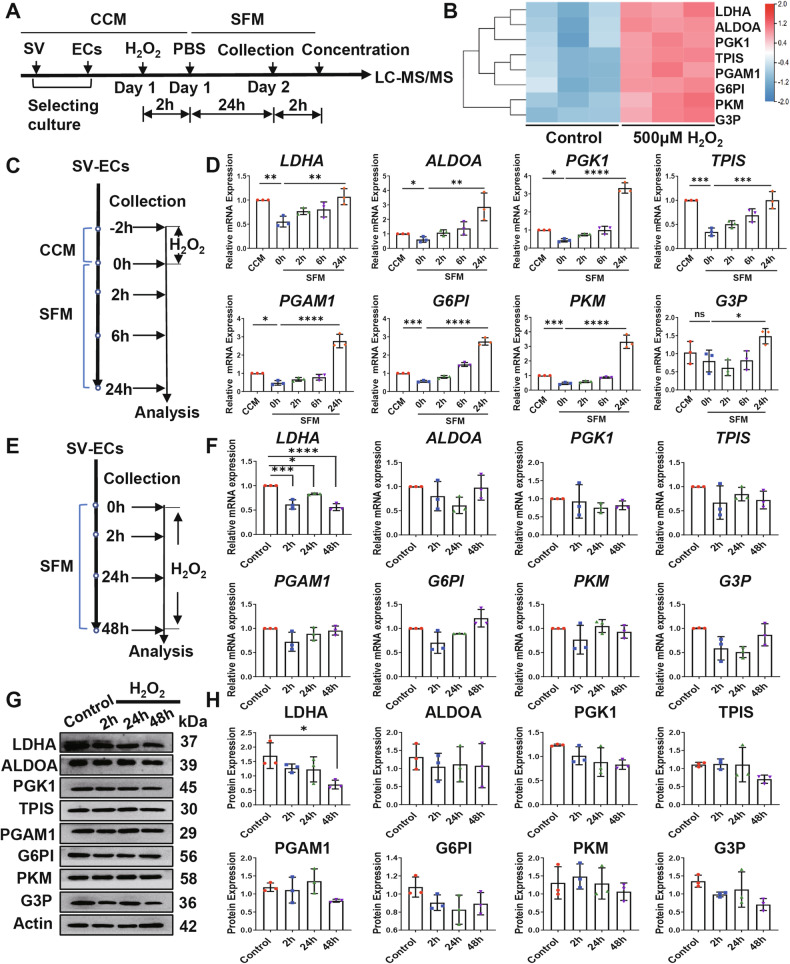
Effects of oxidative stress on glycolysis pathway. **A** Schematic diagram illustrating the preparation of supernatant from SV-ECs following short-term stimulation of 500 μM H_2_O_2_ for 2 h. **B** Heat Map showing the differential expression analysis of SV-ECs treated with or without 500 μM H_2_O_2_. **C**–**F** SV-ECs were cultured in CCM with short-term stimulation of 500 μM H_2_O_2_ for 2 h and subsequently in SFM without H_2_O_2_ for another 24 h (**C**, **D**), or in SFM with long-term stimulation of 500 μM H_2_O_2_ for 48 h (**E**, **F**). Relative mRNA levels of glycolysis-related genes were analyzed by qRT-PCR (**D**, **F**). **G**, **H** Western blot analysis of the glycolysis-related genes in SV-ECs treated with H_2_O_2_ for 2, 24 and 48 h (*n* = 3). The data is expressed as Mean ± SD. *P < 0.05, **P < 0.01, ***P < 0.001, ****P < 0.0001 analyzed by one-way ANOVA. ALDOA Fructose-bisphosphate aldolase A, CCM complete culture medium, ECs endothelial cells, G3P Glyceraldehyde-3-phosphate dehydrogenase, G6PI Glucose-6-Phosphate Isomerase, LC-MS/MS liquid chromatography tandem mass spectrometry; LDHA L-lactate dehydrogenase A chain, ns not significant, PGAM1 Phosphoglycerate mutase 1, PGK1 Phosphoglycerate kinase 1, PKM Pyruvate kinase, qRT-PCR Quantitative Reverse Transcription Polymerase Chain Reaction, SFM serum-free medium; SV stria vascularis, TPIS Triosephosphate isomerase.

Taken together, our results indicated that the function of SV-ECs is impaired following noise-induced oxidative stress.

### Oxidative stress altered glycolysis in SV-ECs in vitro and in vivo

We have previously investigated the differentially expressed proteins in SV-ECs treated with H_2_O_2_ using LC-MS/MS technology [14], as shown in Fig. 2A. Our analysis revealed that the enriched pathways of differential proteins were predominantly associated with glycolysis, including LDHA, ALDOA, PGK1, TPIS, PGAM1, G6PI, PKM, and G3P (Fig. 2B). To elucidate the role of glycolysis in SV-ECs under oxidative stress, cells were exposed to 500 μM H_2_O_2_ for 2 h and subsequently analyzed at various time points using qRT-PCR and Western blot. We observed a significant decrease in the expression of glycolysis-related genes after 2 h of H_2_O_2_ treatment, followed by a gradual increase at 2, 6, and 24 h post-stimulus removal (Fig. 2C, D). However, Western blot analysis showed no significant changes in the levels of all of the glycolysis-related proteins (Supplementary Fig. S2A). Moreover, among these glycolysis-related factors, we found that only the mRNA (Fig. 2E, F) and protein (Fig. 2G, H) expressions of glycolysis-related LDHA were significantly reduced after 48 h of continuous H_2_O_2_ treatment. These data suggested that glycolysis-related LDHA could be involved in the regulation of SV-ECs injuries mediated by oxidative stress.

**Fig. 3:**
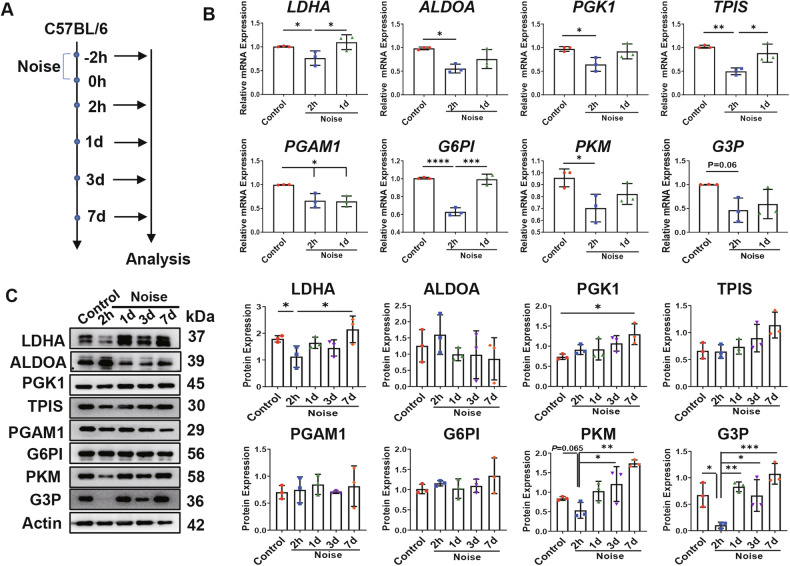
Effects of noise exposure on glycolysis pathway. **A** Schematic diagram illustrating the preparation of SV of cochleae after noise exposure_._ **B** Relative mRNA levels of glycolysis-related proteins in SV of C57BL/6 mice exposed to noise. **C** Western blot analysis of glycolysis pathway proteins in whole cochlear tissue homogenates of noise-exposed and control C57BL/6 mice. The data is presented as Mean ± SD. *P < 0.05, **P < 0.01, ***P < 0.001, ****P < 0.0001 analyzed by one-way ANOVA. ECs endothelial cells, G3P Glyceraldehyde-3-phosphate dehydrogenase, G6PI Glucose-6-Phosphate Isomerase, LDHA L-lactate dehydrogenase A chain, ns not significant, PGAM1 Phosphoglycerate mutase 1, PGK1 Phosphoglycerate kinase 1, PKM Pyruvate kinase, qRT-PCR Quantitative Reverse Transcription Polymerase Chain Reaction, SV stria vascularis, TPIS Triosephosphate isomerase.

To further validate our in vitro findings, we obtained SV tissues from a mouse model of NIHL at various time points post-noise exposure and analyzed them using qRT-PCR and Western blot (Fig. 3A). The results demonstrated a significant decrease in the levels of most glycolysis-related genes at 2 h post-noise exposure, with recovery observed at 1 day post-exposure (Fig. 3B). Similarly, the expression of glycolysis-related proteins, including LDHA, PKM, and G3P, significantly decreased at 2 h post-noise exposure and gradually increased thereafter (Fig. 3C).

Overall, our data indicated that noise-induced oxidative stress rapidly and robustly inhibits the expression of glycolysis-related LDHA in SV-ECs.

### Inhibition of LDHA reduced viability and promoted the anti-oxidative response in SV-ECs

**Fig. 4:**
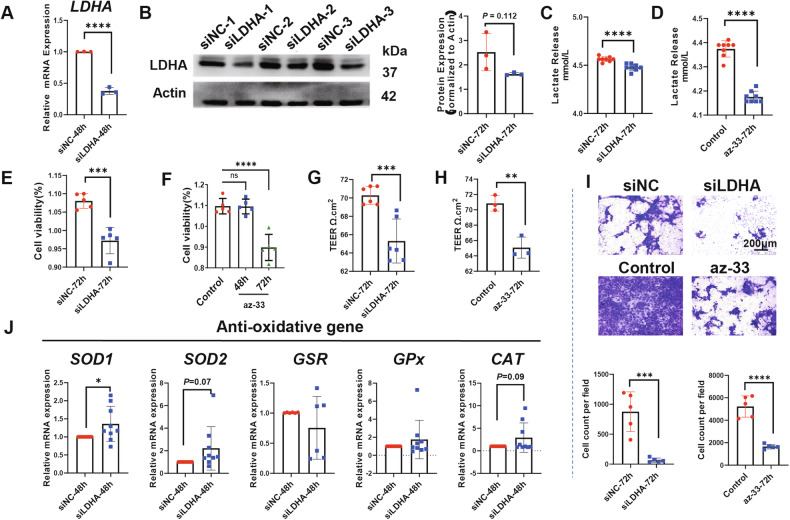
Role of LDHA in SV-ECs function*.* **A** mRNA levels of LDHA were decreased in SV-ECs stimulated by siLDHA. **B** Quantitation and representative image of protein expression of LDHA. **C**, **D** Lactate release were decreased in supernatant of SV-ECs stimulated by siLDHA (50 nM) or az-33 (10 μM) for 72 h. **E**, **F** Cell viability assay for ECs pre-treated with siRNA (50 nM) or az-33. **G**, **H** TEER significantly decreased in control, siLDHA, and az-33 treated groups. **I** Representative images and quantitation of transwell migration assays of SV-ECs (scale bar, 200 μm). **J** Relative mRNA levels of anti-oxidative, including *SOD1*, *SOD2,GSR,CAT and GPx*, were increased in SV-ECs stimulated by siLDHA as demonstrated by qRT-PCR. The data is expressed as Mean ± SD (*n* ≥ 3). *P < 0.05, **P < 0.01, ***P < 0.001, ****P < 0.0001 analyzed by unpaired t test. CAT Catalase, ECs endothelial cells, GSR Glutathione-disulfide reductase, GPx Glutathione peroxidases, LDHA L-lactate dehydrogenase A chain, ns not significant, qRT-PCR Quantitative Reverse Transcription Polymerase Chain Reaction, SOD1 Superoxide Dismutase 1, SOD2 Superoxide Dismutase 2, SV stria vascularis, TEER Transepithelial electrical resistance.

To elucidate the role of LDHA in SV-ECs function, we employed siRNA to reduce LDHA expression, as confirmed by qRT-PCR (Fig. 4A) and Western blot (Fig. 4B). Following LDHA inhibition, lactate released by SV-ECs was significantly reduced (Fig. 4C). To corroborate these findings, we used az-33, a small molecule inhibitor of LDHA, which similarly decreased lactate release in SV-ECs (Fig. 4D). Cell viability assays (CCK8) demonstrated a significant reduction in SV-ECs viability after treatment with both siLDHA and az-33 (Fig. 4E, F). Additionally, LDHA inhibition via siRNA-LDHA and az-33 significantly impaired barrier integrity and migration of SV-ECs (Fig. 4G, H and Fig. 4I, respectively). Furthermore, the mRNA expression of antioxidant genes, including SOD1, SOD2, and CAT, was significantly increased or showed a trend toward statistical significance following LDHA inhibition in SV-ECs by siRNA-LDHA (Fig. 4J). In total, these findings indicated that inhibiting LDHA significantly reduces the viability of SV-ECs while concurrently enhancing their antioxidant function.

### Inhibition of LDHA in SV-ECs exhibited anti-inflammatory effects on Mφ

Previous studies have demonstrated that exposure to intense acoustic overstimulation results in an increase in circulating monocytes, which subsequently enter the cochlea and differentiate into mature Mφ [22]. Consistently, immunofluorescence analysis revealed a significant increase in Mφ (IBa1^+^) within the SV, spiral ganglion neuron (SGNs), modiolus, and spiral ligament of the cochlea at 7 days post-noise exposure (Supplementary Fig. S3A). Additionally, flow cytometry analysis of cochlear Mφ showed an increased proportion of CD86/CD206, though the percentage of pro-inflammatory CD86^+^ Mφ only approached tendency towards significance and no significant difference was observed in the percentages of anti-inflammatory CD206^+^ Mφ (Supplementary Fig. S3B-C).

**Fig. 5:**
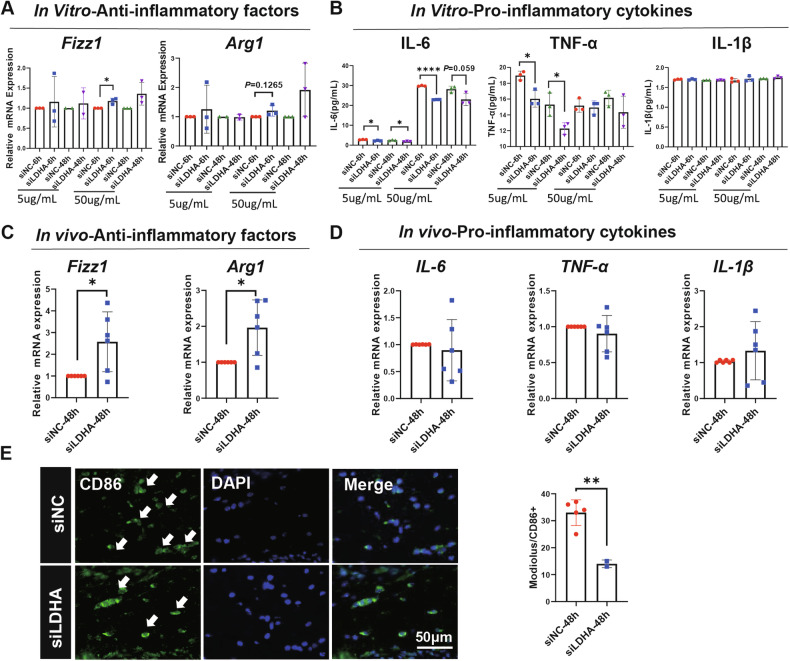
Effects of LDHA inhibition in SV-ECs on Mφ function*.* **A**, **B** Levels of anti-inflammatory cytokines, such as *Fizz1* and *Arg1*, were increased in Mφ following treatment of secretomes of SV-ECs with inhibition of LDHA as determined by qRT-PCR (**A**), while pro-inflammatory cytokines, such as IL-6 and TNF-α, were decreased as demonstrated by ELISA (B) (*n* = 3). **C**, **D** Levels of anti-inflammatory cytokines, such as *Fizz1* and *Arg1*, were increased in cochlea of mice with intra-tympanic administration of siLDHA as demonstrated by qRT-PCR (**C**), while pro-inflammatory cytokines, such as IL-6, TNF-a and IL-1β, remained unchanged (**D**) (*n* = 6). **E** Representative images and analysis of CD86^+^ cells in modiolus of mice with intra-tympanic administration of siLDHA (scale bar, 50 μm). The data is expressed as Mean ± SD. *P < 0.05, **P < 0.01, ****P < 0.0001 analyzed by unpaired *t* test. ELISA enzyme-linked immunosorbent assay, ECs endothelial cells, LDHA L-lactate dehydrogenase A chain, Mφ macrophages, NC normal control, ns not significant, qRT-PCR Quantitative Reverse Transcription Polymerase Chain Reaction, SV stria vascularis.

To further elucidate the role of LDHA in the immunoregulation of SV-ECs on Mφ differentiation, we first stimulated Mφ with the secretome of SV-ECs treated with either siNC or siLDHA in vitro. Notably, the level of anti-inflammatory factor *Fizz1* was significantly increased at 6 h in the presence of secretomes of siLDHA-treated SV-ECs, as demonstrated by qRT-PCR (Fig. 5A). Consistently, levels of proinflammatory factors IL-6 and TNF-α in the supernatants were significantly decreased in Mφ stimulated with siLDHA-treated SV-ECs secretomes using ELISA (Fig. 5B). Furthermore, we investigated the role of LDHA in regulating cochlear Mφ function in vivo. We found that intra-tympanic administration of siLDHA significantly inhibited LDHA expression, as shown by qRT-PCR and Western blot analyses (Supplementary Fig. S4A-D). This inhibition led to a significant increase in the levels of anti-inflammatory factors *Fizz1* and *Arg1*, as demonstrated by qRT-PCR (Fig. 5C), while levels of pro-inflammatory cytokines IL-6, TNF-α, and IL-1β remained unaffected (Fig. 5D). Additionally, the level of pro-inflammatory CD86^+^ Mφ in the modiolus was significantly decreased following intra-tympanic administration of siLDHA (Fig. 5E). Overall, our data indicated that LDHA exerts pro-inflammatory effects on Mφ both in vitro and in vivo.

### LDHA modulates SV-ECs-mediated dysfunction of Mφ through CX3CL1-CX3CR1 pathway

**Fig. 6:**
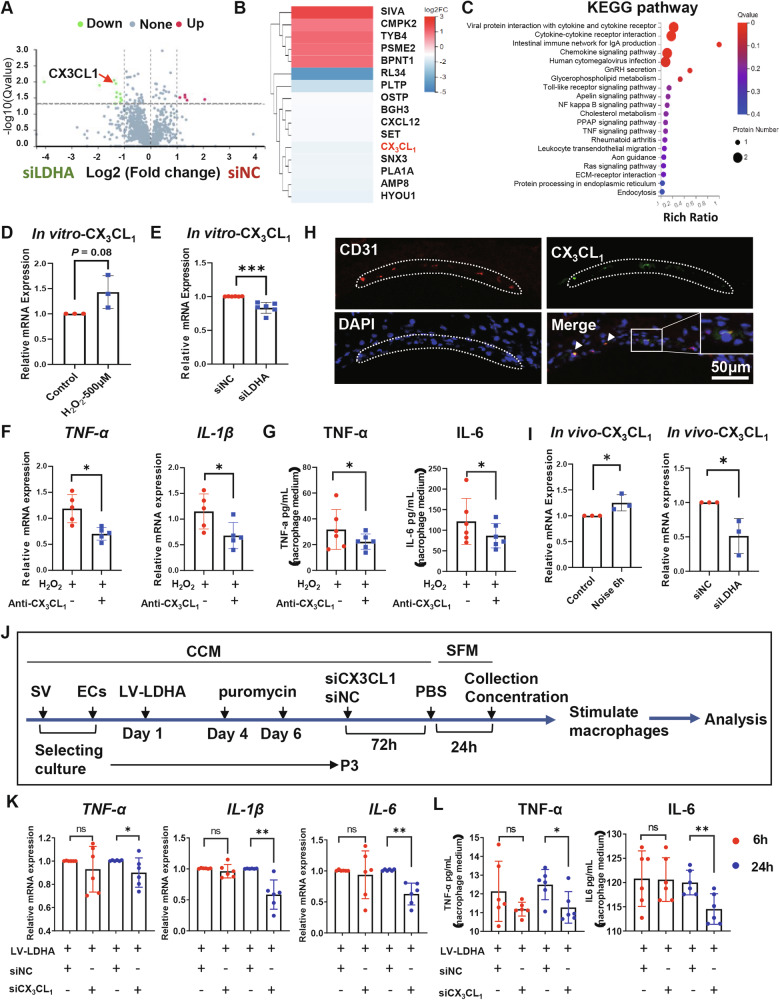
LDHA inhibition in SV-EC mediated anti-inflammatory effects on Mφ via CX3CL1-CX3CR1 signaling pathway. **A**–**C** LC-MS/MS analysis of supernatants of SV-ECs treated with siNC or siLDHA for 72 h. **A** Volcano plots for the differential expression analysis of secretome released by SV-ECs treated with siNC or siLDHA as indicated. **B** Heat map displaying differential protein expression between SV-ECs treated with siLDHA and siNC. **C** KEGG pathway analysis illustrating the function of the differential secretome between control and siLDHA-treated SV-ECs. **D**, **E** Relative mRNA levels of CX3CL1 in SV-ECs stimulated by 500 μM H_2_O_2_ for 2 h (**E**) or siLDHA for 48 h (**F**) in vitro, as demonstrated by qRT-PCR. **F** Levels of pro-inflammatory cytokines, such as TNF-α and IL-1β, were decreased in H_2_O_2-_treated Mφ (2 h) following treatment of CX3CL1 neutralizing antibody for 6 h, as demonstrated by qRT-PCR (*n* = 5). **G** Levels of pro-inflammatory cytokines, such as *IL-6* and *TNF-α*, were decreased in secretomes of H_2_O_2_-treated Mφ (2 h) stimulated with CX3CL1 neutralizing antibody for 6 h as demonstrated by ELISA (*n* = 6). **H** Representative confocal images showing co-expression of CX3CL1 (green) and CD31 (red) in the endothelial cells of the SV (scale bar, 50 μm). **I** Relative mRNA levels of CX3CL1 in cochlea of noise-exposed mice (2 h) compared to control mice, and in noise-exposed mice (2 h) with or without intratympanic administration of siLDHA in vivo after 6 h, as demonstrated by qRT-PCR. **J** Schematic diagram illustrating the preparation of the secretomes from SV-ECs with overexpression of LDHA by lentivirus transfection and inhibition of siCX3CL1 by siCX3CL1treatment. **K** Levels of pro-inflammatory cytokines, including *TNF-α*, *IL-1β*, and *IL-6*, were significantly decreased in the mRNA levels of Mφ treated with the secretomes from LV-LDHA-siCX3CL1-SV-ECs at 24 h, as demonstrated by qRT-PCR (*n* = 6). **L** Levels of pro-inflammatory cytokines, such as *IL-6* and *TNF-α*, were decreased in supernatant of Mφ stimulated with the secretomes from LV-LDHA-siCX3CL1-SV-ECs at 24 h, as demonstrated by ELISA (*n* = 6). The data is expressed as Mean ± SD. *P < 0.05, **P < 0.01, ***P < 0.001 analyzed by unpaired *t* test or Mann–Whitney test. CCM complete culture medium, ECs endothelial cells, LDHA L-lactate dehydrogenase A chain, LV lentivirus, NC normal control, qRT-PCR Quantitative Reverse Transcription Polymerase Chain Reaction, SFM serum-free medium, SV stria vascularis, ns not significant.

We first performed proteomics analysis to investigate the difference between secretomes released by SV-ECs stimulated with siNC and siLDHA. In total, we identified 16 differential proteins with the criteria of a multiple of difference >2 and a *P* < 0.05 (Fig. 6A, B). The 16 differential proteins in SV-ECs treatment with siLDHA and siNC were mainly involved in pathways such as viral protein interaction with cytokine and cytokine receptor, cytokine-cytokine receptor interaction, intestinal immune network for IgA production, and chemokine signaling pathway according to the KEGG analysis (Fig. 6C).

Previous studies have demonstrated that chemokines such as CCL2, CCL4, and CXCL12 are pivotal in mediating the inflammatory response within the cochlea, which contributes to hearing loss [31, 32]. In our study, we observed that expression of *CX3CL1* was elevated in SV-ECs treated with 500 μM H_2_O_2_ and subsequently decreased upon inhibition of LDHA in SV-ECs, as shown by qRT-PCR in vitro (Fig. 6D, E). Furthermore, mRNA expressions of inflammatory factors in Mφ, including *TNF-α* and *IL-1β*, were significantly reduced following neutralization of CX3CL1 in the secretomes of H_2_O_2_-treated SV-ECs (Fig.6F). Similarly, ELISA indicated that TNF-α and IL-6 levels (with *IL-1β* being undetectable) were significantly reduced in the supernatants of Mφ treated with secretomes from SV-ECs after CX3CL1 neutralization (Fig. 6G). Immunofluorescence analysis revealed the presence of CX3CL1 in the SV-ECs of NIHL mice (Fig. 6H), suggesting that SV-ECs may be a significant source of CX3CL1. Additionally, we found that CX3CL1 expression was increased in the cochlea following noise exposure and decreased after intra-tympanic administration of siLDHA, as demonstrated by qRT-PCR (Fig. 6I).

To further investigate the association between LDHA and CX3CL1 in SV-ECs, we established LDHA-overexpressing SV-ECs by using lentivirus transfection, as confirmed by fluorescence imaging (Supplementary Fig. S5A-B). Subsequently, CX3CL1 expression was silenced via siRNA targeting CX3CL1, as validated by qRT-PCR (Supplementary Fig. S5C) and Western blot analysis (Supplementary Fig. S5D). The secretomes from LDHA-overexpressing SV-ECs treated with siCX3CL1 were collected and applied to Mφ cultures for 6 and 24 h (Fig. 6J). We observed that the mRNA levels of inflammatory factors, including *TNF-α*, *IL-1β*, and *IL-6*, were significantly reduced in Mφ at 24 h (Fig. 6K). Similarly, ELISA revealed a significant decrease in the secretion of TNF-α and IL-6 (with IL-1β being undetectable) in the supernatants of Mφ at 24 h (Fig. 6L).

Collectively, these findings indicated that CX3CL1-CX3CR1 pathway was implicated in the proinflammatory effects of SV-ECs on Mφ mediated by LDHA.

## Discussion

In our current study, we discovered that oxidative stress induced by H_2_O_2_ and noise leads to functional damage to SV-ECs during the acute phase, both in vitro and in vivo. Notably, for the first time, we revealed that the expression of glycolysis-related proteins was significantly altered in H_2_O_2_-stimulated SV-ECs and in mouse SV following noise-induced oxidative stress injury, with the reduction of LDHA at the early phase being the most pronounced. Subsequently, we found that inhibition of LDHA played a crucial role in oxidative stress-induced SV-ECs injury while mediating the anti-inflammatory effects on Mφ. Furthermore, we discovered that glycolysis-related LDHA mediated the interaction between SV-ECs and Mφ through the CX3CL1-CX3CR1 signaling pathway.

SV-ECs have been intensively reported to play a critical role in maintaining the integrity of the blood-labyrinth barrier (BLB) in inner ear [33, 34]. Notably, SV-ECs, which are distributed throughout the microvascular system within SV and restrict intercellular channels, are believed to be essential for maintaining the balance between the endolymph and perilymph [35, 36]. Recent studies have found novel and significant roles of SV in hearing loss. For instance, in age-related hearing loss, the upregulation of heat shock protein 90AA1 (Hsp90AA1) has been reported to mitigate endoplasmic reticulum stress damage in SV cells [30]. Also, the SV has been identified as a primary site of age-related cochlear degeneration and Mφ dysfunction associated with age-related hearing loss in both mice and humans [37]. However, to our knowledge, the role of SV-ECs in response to noise-induced oxidative stress has rarely reported. In this study, we isolated SV-ECs as previously reported [14, 29] and demonstrated that oxidative stress induced by H_2_O_2_ impairs the migration and angiogenic capabilities of SV-ECs in a dose-dependent manner. We also observed that SV-ECs produced excessive reactive oxygen species (ROS) and exhibited decreased expression of antioxidant genes. This observation is consistent with previous reports indicating reduced activity of antioxidant enzymes in the cochleae of noise-exposed mice [38, 39]. This could represent a negative feedback mechanism in SV-ECs, where antioxidant proteins are excessively consumed to counteract the overproduction of oxygen free radicals, thereby maintaining the oxidative balance in the cochlea. These findings prompted us to further investigate the cellular mechanisms underlying SV-ECs responses to oxidative stress.

Furthermore, we investigated the possible cellular mechanisms of oxidative stress-induced SV-ECs by first identifying the differential expression pathways in these cells. Interestingly, we found that the glycolysis pathway was one of the enriched pathways related to oxidative stress in SV-ECs. These results led us to hypothesize that glycolysis in SV-ECs might mediate oxidative stress responses. To confirm our hypothesis, we further examined the differential gene expression of glycolysis-related genes in SV-ECs induced by oxidative stress. As expected, we observed that expression of the most of the glycolysis-related genes was significantly decreased compared to the control after 2 h of H_2_O_2_ treatment, while the protein expression remained unchanged. We speculated that the duration of H_2_O_2_ stimulation for 2 h may be insufficient to affect protein levels. Therefore, we extended the H_2_O_2_ stimulation to 24 and 48 h, and both gene and protein levels of glycolysis-related proteins showed a downward trend. Notably, the gene and protein expression levels of LDHA significantly decreased at 2 and 48 h after stimulation, respectively. Similarly, the levels of glycolysis-related genes in SV were decreased at 2 h after noise exposure in vivo, alongside with the decreases on the protein levels of LDHA, pyruvate Kinase M (PKM), and glyceraldehyde 3-phosphate (G3P) at 2 h post-noise exposure. These data suggested intracellular generation of ROS can inhibit glycolysis-related LDHA during oxidative stress at early phase. While the effects of noise-induced oxidative stress on hair cells are gradual and sustained [5, 40], our findings demonstrated that its impact on the SV, particularly on SV-ECs, is both rapid and pronounced, suggesting that SV-ECs could possibly be the early injury site as previously reported in age-related hearing loss [37]. Also, our findings are consistent with previous studies, which showed that inhibition of glycolysis allows cells to redirect metabolic flux to the oxidative pentose phosphate pathway to promote NADPH synthesis and prevent oxidative stress responses [41]. Based on both the in vitro and in vivo experiments, we demonstrated that glycolysis-related LDHA is consistently a downregulated protein. Therefore, we propose that glycolysis plays a crucial role in oxidative stress-induced injury of SV-ECs, with LDHA being a key regulatory factor.

LDH is the enzyme that catalyzes the forward and backward conversion of pyruvate to lactate. LDHA converts pyruvate to lactate and produces nicotinamide adenine dinucleotide (NAD+). On the contrary, LDHB catabolizes lactate to pyruvate. Previous studies have indicated that targeting LDH presents an attractive strategy for the development of therapies for sensorineural hearing loss [17]. However, there is currently no relevant report on the effect of LDHA on oxidative stress damage in the cochlea. Oxidative stress can cause SV-ECs damage and lead to a decrease in LDHA. Conversely, inhibition on LDHA can promote the antioxidant response of SV-ECs. This may represent a negative feedback mechanism in SV-ECs following oxidative stress damage, which suggested that inhibition of LDHA was able to exhibit protective effects on noise-induced oxidative injury to SV. Based on this evidence, we hypothesized that factors released from SV-ECs lacking LDHA might also directly regulate the bystander cells such as Mφ in response to oxidative stress.

Dysfunction of inner ear Mφ have been demonstrated to play a crucial role in the development of NIHL [24, 42]. Therefore, we further investigated the effects of LDHA inhibition in SV-ECs on inner ear Mφ and the underlying mechanisms. We examined the differential proteins released by SV-ECs with or without LDHA inhibition using the in vitro serum-free SV-ECs culture method we previously established [14]. Our data indicated that the secretomes of SV-ECs treated with siLDHA was enriched in cytokines and cytokine receptors, including CX3CL1 and CCL12. CX3CL1, also known as Fractalkine, is primarily secreted by endothelial cells and neurons [43], and is closely associated with the pathogenesis of inflammatory diseases such as atherosclerosis, renal fibrosis, rheumatoid arthritis, and both acute and chronic neurodegenerative diseases of the central nervous system (CNS) [44]. Recent studies have highlighted the role of LDHA in facilitating regulatory T cells through lactate and GPR81 via the regulation of CX3CL1 [45]. In our study, we demonstrated that neutralizing CX3CL1 antibody effectively attenuates the pro-inflammatory effects of secretomes collected from H_2_O_2_-treated SV-ECs on Mφ. Additionally, the secretomes LDHA-overexpressing SV-ECs subjected to siCX3CL1 treatment also exhibited reduced pro-inflammatory effects on Mφ. These findings suggest that LDHA plays a critical role in mediating the release of CX3CL1 from SV-ECs. CX3CL1 signal through its sole receptor, CX3CR1, which is located on microglial cells and Mφ. Previous studies have predominantly focused on the interaction between cochlear SGNs and Mφ regarding the CX3CL1-CX3CR1 signaling pathway [46, 47]. For the first time, our data revealed that inhibition of LDHA in SV-ECs exhibited anti-inflammatory effects on Mφ through the CX3CL1-CX3CR1 signaling pathway.

We acknowledged that there are some limitations in our study. While our findings demonstrated that LDHA inhibition in SV-ECs confers significant anti-oxidative stress and anti-inflammatory effects, further validation through in vivo experiments using mouse models with specific knockdown of LDHA in SV-ECs is required to substantiate these results. Additionally, our focus was primarily on the direct effects of the secretome on the function of inner ear Mφ. It is possible that the secretome also exerts indirect effects on other cells, such as SGNs and hair cells. Moreover, although qRT-PCR and Western blot were effective in identifying the cochlear Mφ phenotypes, employing more advanced techniques such as flow cytometry and multiplex immunohistochemistry would provide a more comprehensive understanding of inner ear Mφ. Therefore, further research is needed to fully address these issues.

## Conclusions

In this study we discovered that oxidative stress causes acute functional damage to SV-ECs both in vitro and in vivo and a marked reduction in glycolysis-related LDHA during the early phase. Also, we observed that inhibition of LDHA in SV-ECs mediated SV-ECs injuries and exhibited anti-inflammatory effects of SV-ECs on Mφ via the CX3CL1-CX3CR1 signaling pathway. Our study suggested that LDHA in SV-ECs could be a novel therapeutic target for the treatment of NIHL.

## Supplementary information

Supplementary figures and table

Original WB data

## Data Availability

Data will be made available on request.
