# Supplementary material for: LDHA-mediated glycolysis in stria vascularis endothelial cells regulates macrophages function through CX3CL1-CX3CR1 pathway in noise-induced oxidative stress
Source: Cell Death Dis. 2025 Feb 3;16(1):65. doi: 10.1038/s41419-025-07394-6 (PMC11791080; doi:10.1038/s41419-025-07394-6)
Supplement: Supplementary file 1 — Supplementary figures and table [file 41419_2025_7394_MOESM1_ESM.pdf]

Figure S1

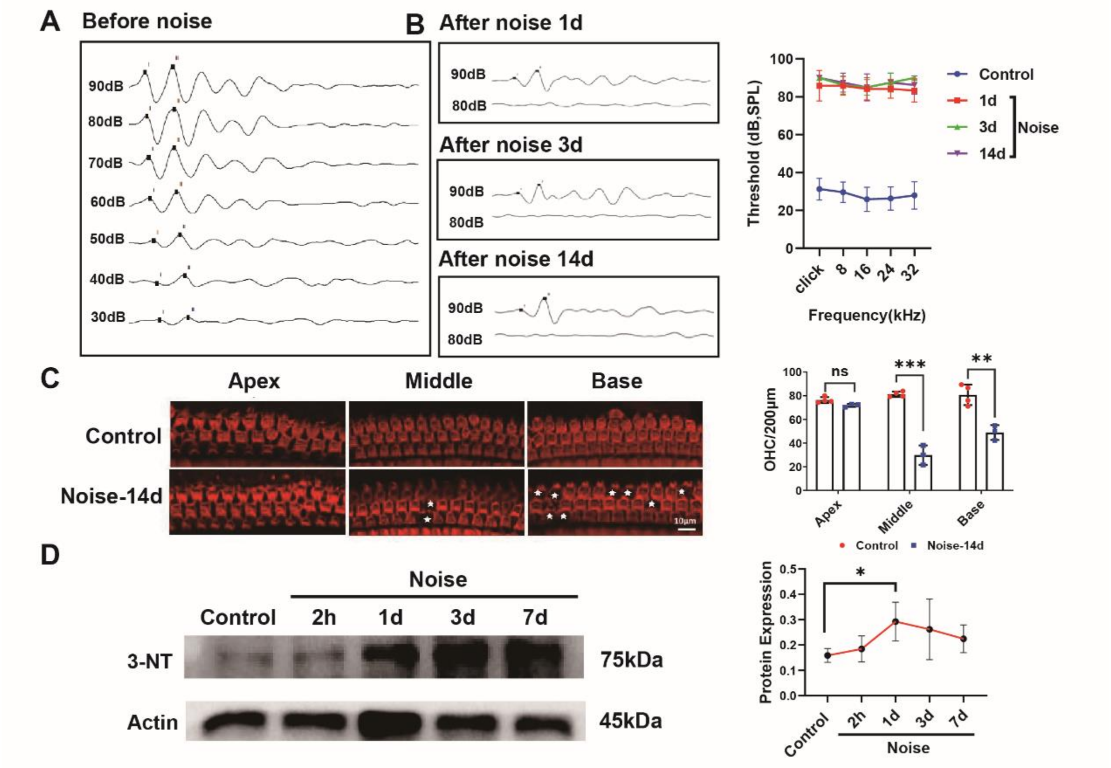

Fig.S1 Noise-mediated oxidative stress damage on hair cells is a slow process. (A-B) ABR threshold and representative ABR waveforms in mice exposed to noise and controls. (**C**) Representative images immunolabeling for OHCs (red) and counts of OHCs of the cochlear surface preparations from mice exposed to noise and controls (scale bar,10μm). The white asterisks represent missing hair cells. (**D**) Western blots analysis of 3-NT, an indicator of oxidative stress, in cochlear tissues of C57BL/6 mice exposed to noise and controls. The data are expressed as Mean ± SD (n = 3). *P< 0.05, **P < 0.001, ***P < 0.001 analyzed by Unpaired t test. OHC, outer hair cell. *3-NT* 3-nitrotyrosine.

Figure S2

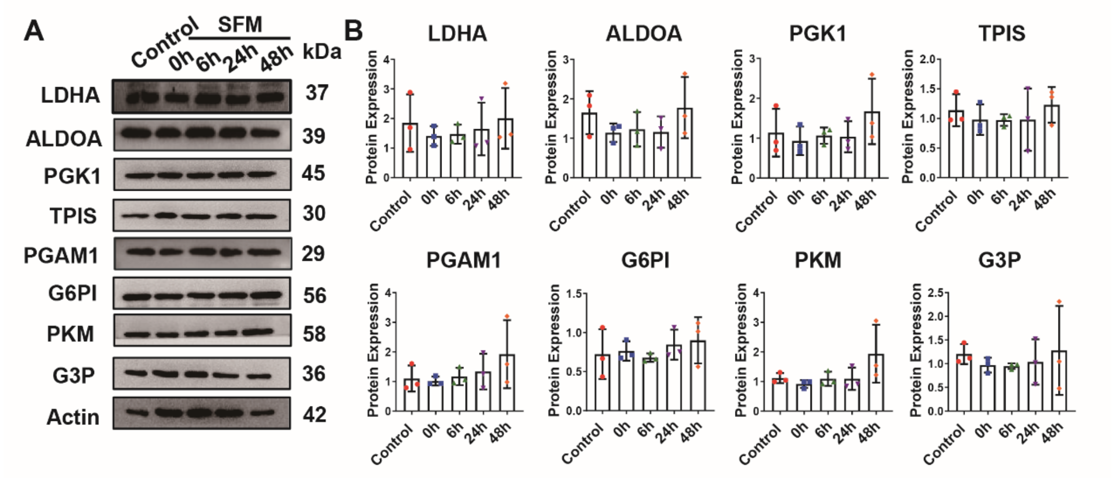

Fig.S2 Effects of short-term stimulation with H_2O_2 on glycolysis pathway in SV-ECs. (A-B) Western blots analysis of glycolysis-related proteins in SV-ECs following short-term stimulation with 500μM H2O2 for 2 hours and subsequent cultured with SFM subsequently for 0, 6, 24 and 48 hours. The data is expressed as Mean ± SD (n = 3) and analyzed by one-way ANOVA. *ALDOA* Fructose-bisphosphate aldolase A, *ECs* endothelial cells, *G3P* Glyceraldehyde-3-phosphate dehydrogenase, *G6PI* Glucose-6-Phosphate Isomerase, *LDHA* L-lactate dehydrogenase A chain, ns not significant, *PGAM1* Phosphoglycerate mutase 1, *PGK1* Phosphoglycerate kinase 1, *PKM* Pyruvate kinase, *SFM* serum-free medium; *SV* stria vascularis, *TPIS* Triosephosphate isomerase.

Figure S3

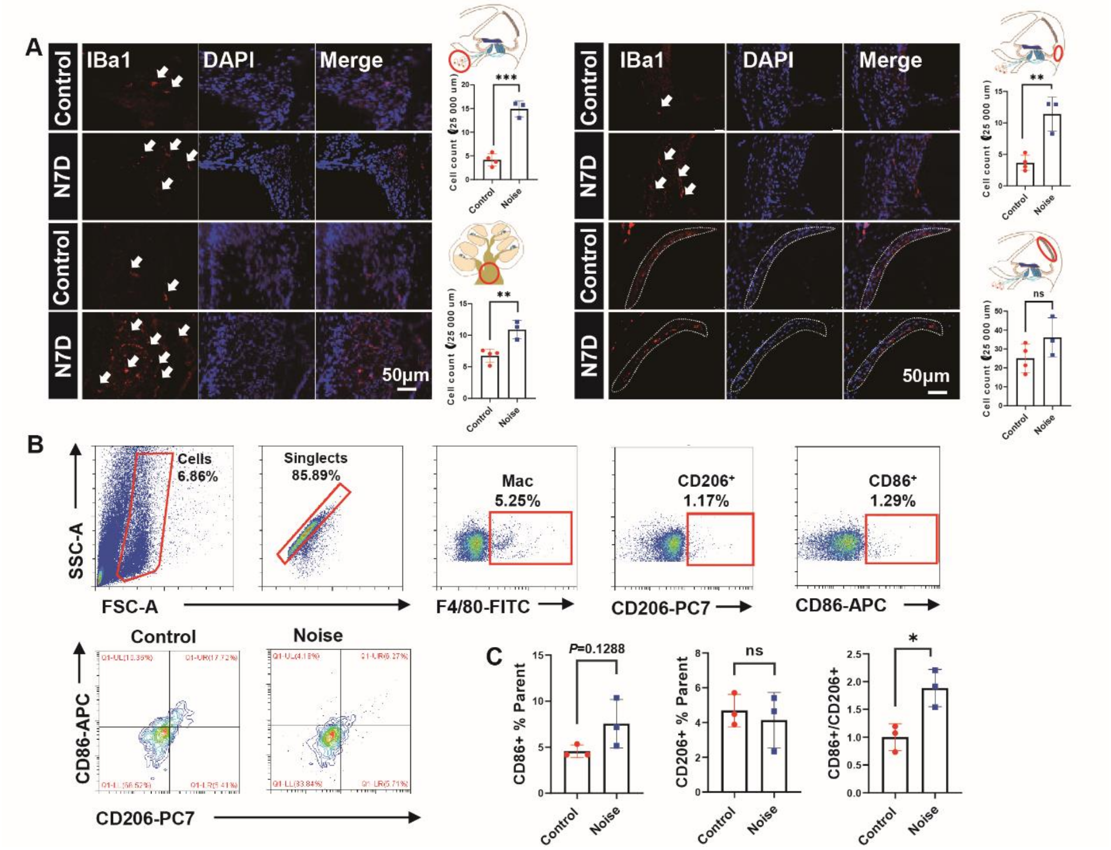

Fig.S3 The effects of noise exposure on the distribution and polarization of inner ear Mφ. (A) Representative images and counts of IBa1-labeled Mφ were increased in the part of SGNs, SL, SV, and modiolus at 7 days after noise exposure (scale bar,50μm). **(B)** Representative dot plots for flow cytometry analysis of the expression of $CD86^+$ and $CD206^+$ Mφ in cochlear cells. **(C)** Percentage of $CD86^+/CD206^+$ Mφ ($F4/80^+$) was significantly increased at 3 days in cochlea of noise-exposed mice (n=3). The data is expressed as Mean ± SD. * $P < 0.05$ analyzed by unpaired t test. SV stria vascularis; *SGN* Spiral ganglion neuron; *SL* Spiral ligament; *Mφ* macrophages.

Figure S4

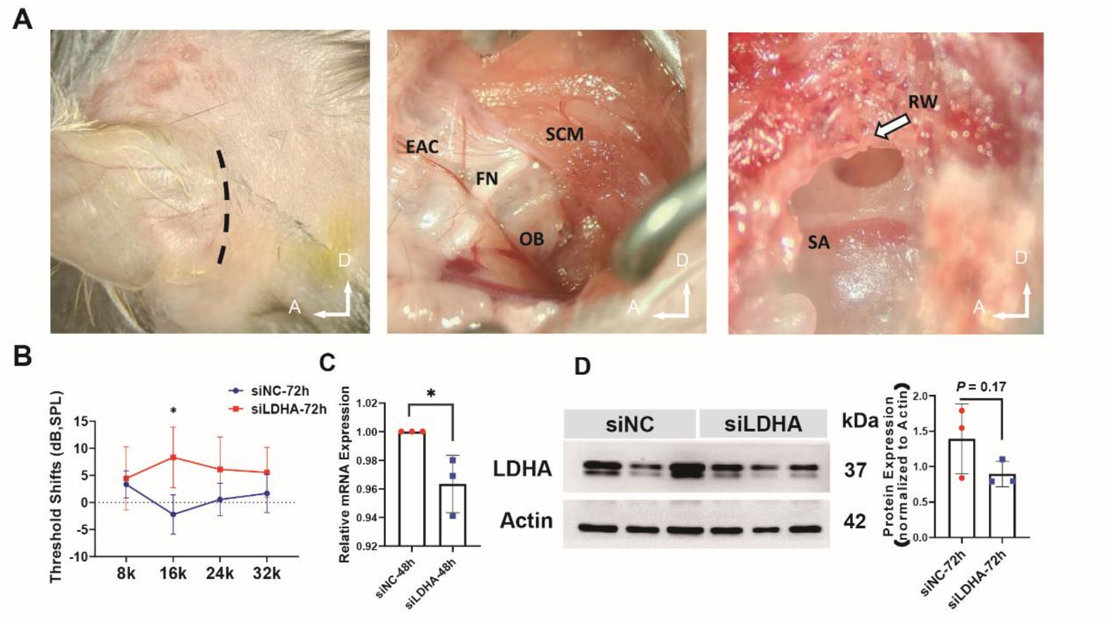

Fig.S4. Effects of intra-tympanic injection of siLDHA on cochlear function and hearing in mice. (A) A retroauricular incision, approximately 2 cm in length and 2 mm away from the external auditory canal, was made as indicated. Magnified views of the operative area show the otic bulla and facial nerve following dermal and subcutaneous incisions to separate the muscle. A 30G needle was used to create a hole in the thin part of the etic bulla (arrow), located ventrally to the facial nerve. Ten microliters of an siRNA-containing solution were delivered to the tympanic cavity and infiltrated into the round window through the hole using a customized catheter. (**B)** Threshold shifts of ABR recorded in mice injected with siLDHA compared to normal controls (n = 9). **(C-D)** Quantitation and representative images of protein and mRNA expression of LDHA in mice treated with siLDHA for 48 hours as determined by qRT-PCR (C) and Western blot (D). The data is expressed as Mean ± SD. *$P < 0.05$ analyzed by Unpaired *t* test, *EAC* external auditory canal, *ECs* endothelial cells, *FN* facial nerve, *LDHA* L-lactate dehydrogenase A chain, *NC* normal control, *OB* otic bulla, *SA* Stapedial artery, *SCM* Sternocleidomastoid-Muscle, *SV* stria vascularis.

Figure S5

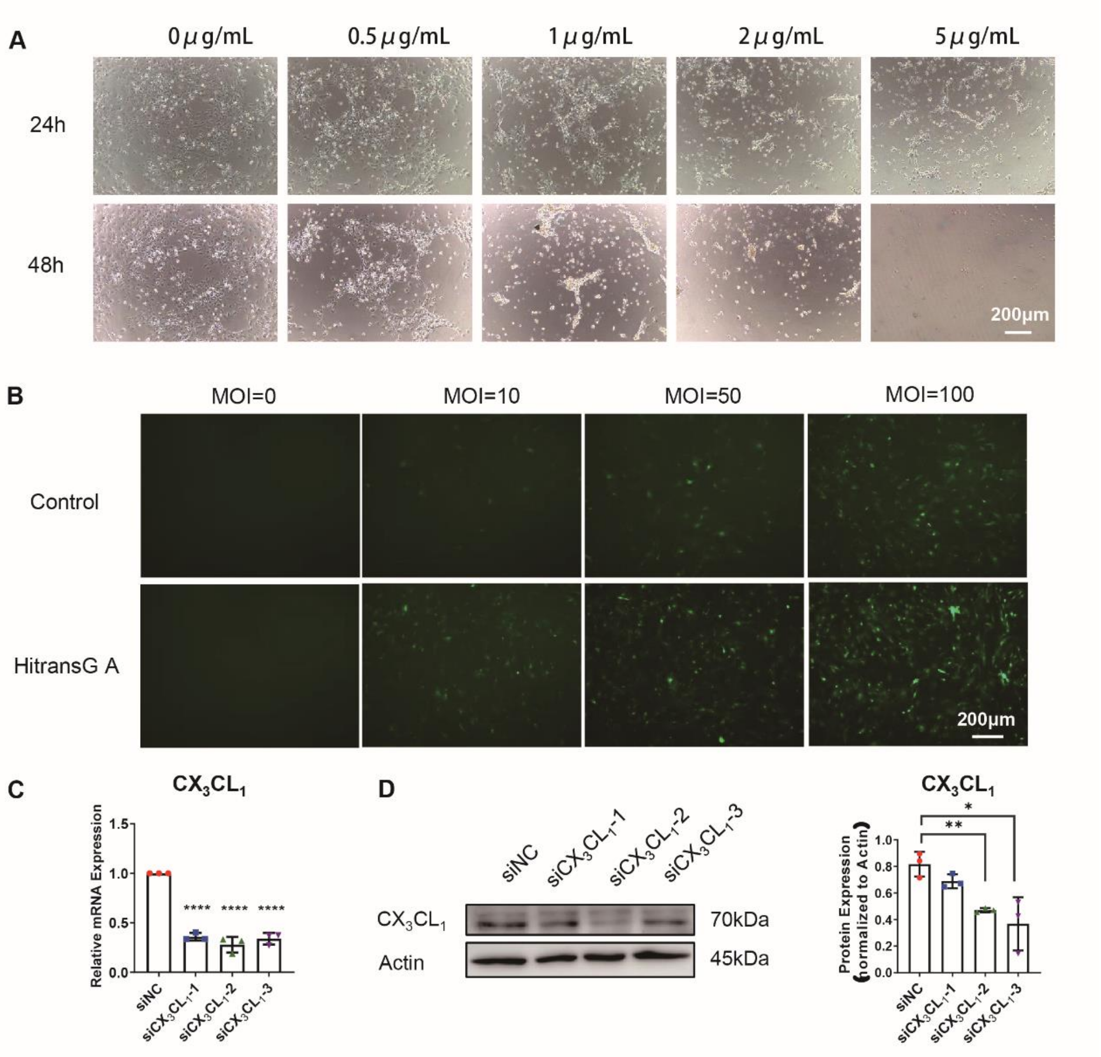

Fig.S5. Establishment of SV-ECs with overexpression of LDHA and knockdown of CX3CL1. (A) SV-ECs were treated with different concentrations of puromycin for 24 hours and 48 hours. Bright-field microscopy showed that nearly all SV-ECs were eliminated after 48 hours of treatment with 1 μg/mL puromycin (scale bar,200μm). **(B)** SV-ECs in a 96-well plate were transduced with different volumes of GFP lentiviral particles and treated with or without HitransG A (an infection enhancement fluid). Expression of GFP was checked by fluorescence microscopy 3 days after transduction, which shows that adding HitransG A and MOI reach to 50 results in high fluorescence

intensity and infection efficiency. Fluorescence microscopy showed that adding HitransG A and increasing the MOI to 50 provided the optimal conditions for infection. (scale bar,200μm). (**C-D)** Quantitation and representative images of mRNA and protein levels of CX3CL1 in SV-ECs treated with siCX3CL1(50nM) for 72 hours as determined by qRT-PCR (C) and Western blot (D), respectively. The data is expressed as Mean ± SD. * $P < 0.05$, ** $P < 0.01$ and **** $P < 0.0001$ analyzed by unpaired t test, *GFP* green fluorescent proteins; *MOI* multiplicity of infection; *SV-ECs* endothelial cells of stria vascularis.

Table S1. Primers for antioxidant genes, inflammatory genes, and glycolysis pathway.

	Forward primer	Reverse Primer
actin	GAGGTATCCTGACCCTGAAGTA	CACACGCAGCTCATTGTAGA
SOD1	GGGTTCCACGTCCATCAGTA	GGTCTCCAACATGCCTCTCT
SOD2	TAACGCGCAGATCATGCAGCTG	AGGCTGAAGAGCGACCTGAGTT
GSR	GCGTGAATGTTGGATGTGTACC	GTTGCATAGCCGTGGATAATTTC
GPx	CGCTCTTTACCTTCCTGCGGAA	AGTTCCAGGCAATGTCGTTGCG
CAT	CCTCGTTCAGGATGTGGTTT	TCTGGTGATATCGTGGGTGA
TNF-α	ATGAGCACAGAAAGCATGA	AGTAGACAGAAGAGCGTGGT
IL-1β	AACCTGCTGGTGTGTGACGTTC	CAGCACGAGGCTTTTTTGTTGT
IL-6	CCTCTCTGCAAGAGACTTCCAT	AGTCTCCTCTCCGGACTTGT
Arg1	ATGAAGAGCTGGCTGGTGTG	GCCAGAGATGCTTCCAACTG
Fizz1	CCCTCCACTGTAACGAAGACTC	CACACCCAGTAGCAGTCATCC
LDHA	GCTCCCCAGAACAAGATTACAG	TCGCCCTTGAGTTTGTCTTC
ALDOA	TTCAGGCTCTTTCCCATCAC	TGGAAGGGATGGCAGATTTAG
PGK1	AACCTCCGCTTTCATGTAGAG	GACATCTCCTAGTTTGGACAGTG
TPIS	TCGAGCAAACCAAGGTCATC	GCTTCTCGTGTACTTCCTGTG
PGAM1	CCCCTTCTACAGCAACATCAGC	GCTCTGGCAATAGTGTCCTTCAG
G6PI	CCATCAAGGTGGACGGCAAAGA	CCGTGATGGATTTGCCAGTGTAC
PKM	CAGAGAAGGTCTTCCTGGCTCA	GCCACATCACTGCCTTCAGCAC
G3P	AGGCCGGTGCTGAGTATGTC	TGCCTGCTTCACCACCTTCT
CX3CL1	CAGTGGCTTTGCTCATCCGCTA	AGCCTGGTGATCCAGATGCTTC
