# Supplementary material for: LDHA-mediated glycolysis in stria vascularis endothelial cells regulates macrophages function through CX3CL1-CX3CR1 pathway in noise-induced oxidative stress
Source: Cell Death Dis. 2025 Feb 3;16(1):65. doi: 10.1038/s41419-025-07394-6 (PMC11791080; doi:10.1038/s41419-025-07394-6)
Supplement: Supplementary file 2 — Original WB data [file 41419_2025_7394_MOESM2_ESM.pdf]

Fig2G-Western blot analysis of the glycolysis-related genes in SV-ECs treated with H_2O_2 for 2, 24 and 48 hours

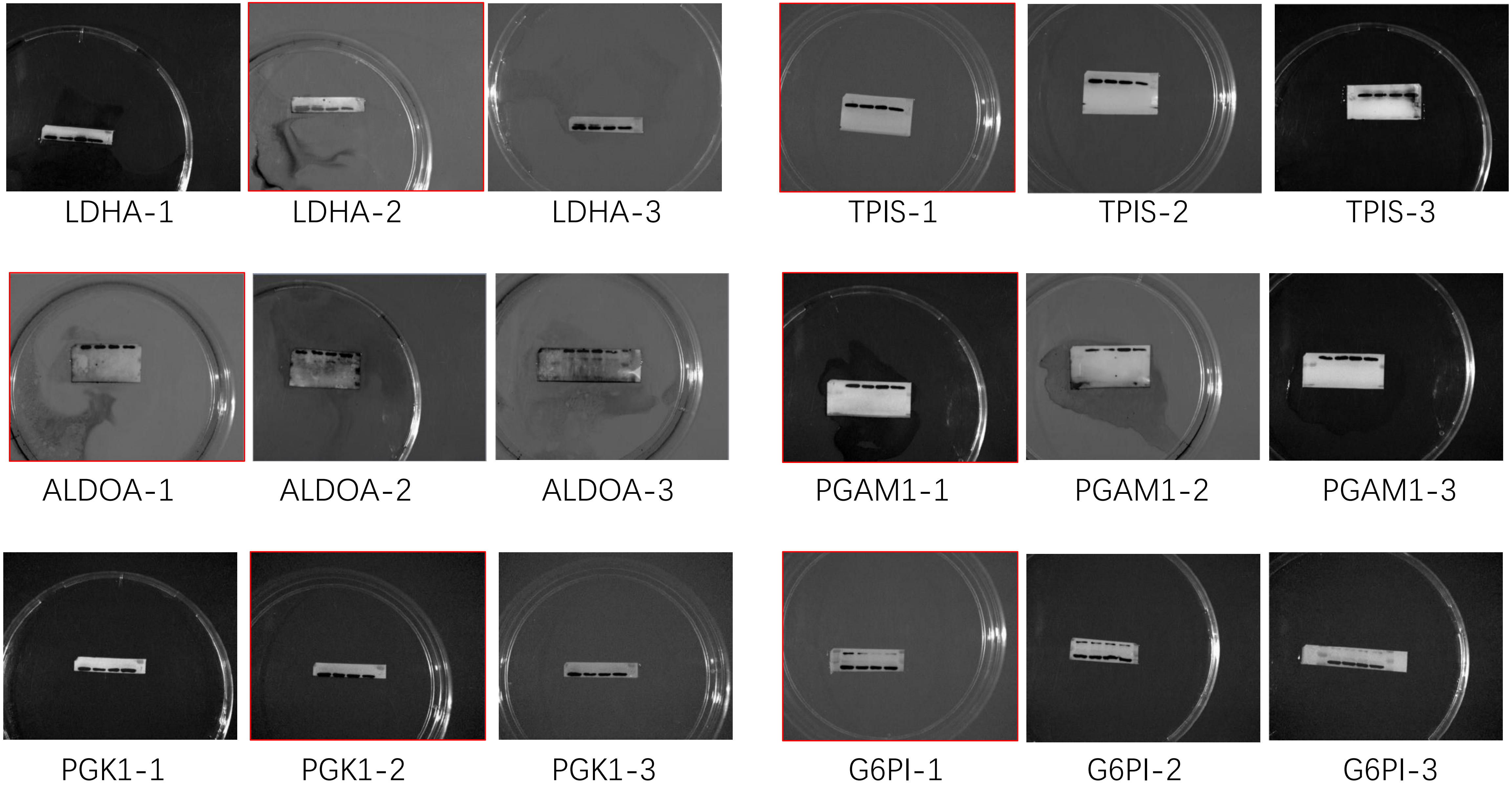

Fig2G-Western blot analysis of the glycolysis-related genes in SV-ECs treated with H_2O_2 for 2, 24 and 48 hours

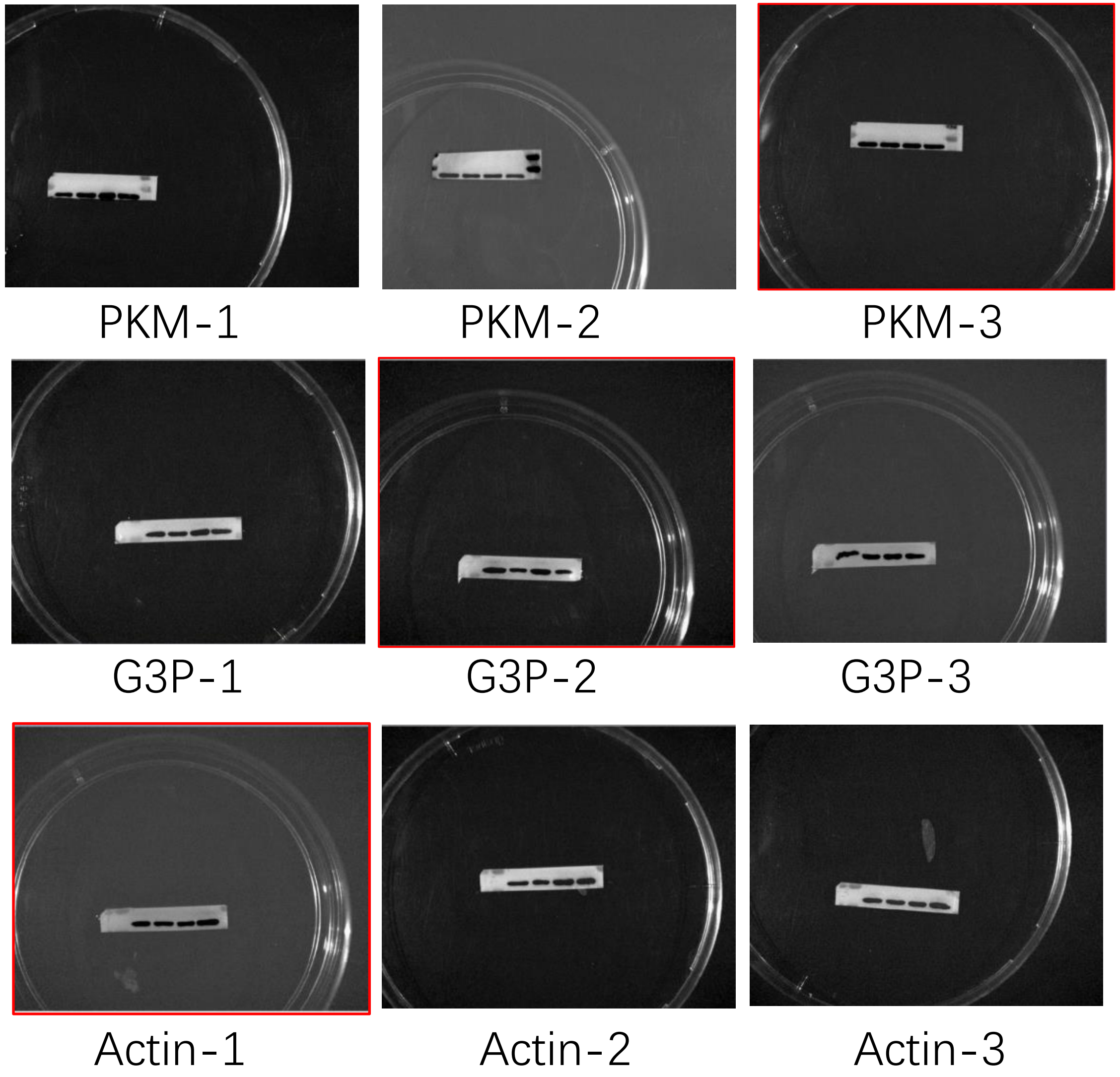

Fig3C-Western blot analysis of glycolysis pathway proteins in whole cochlear tissue homogenates of noise-exposed and control C57BL/6 mice.

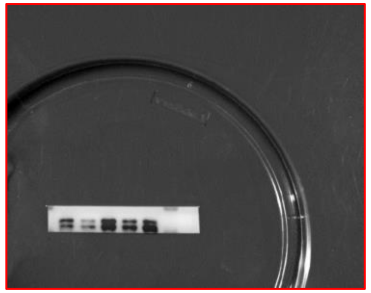
LDHA-1

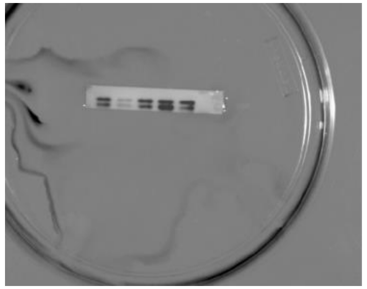
LDHA-2

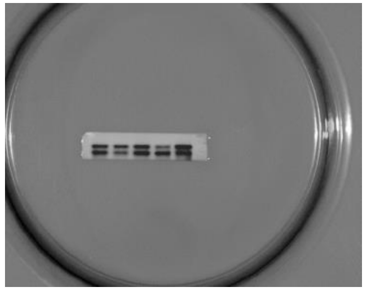
LDHA-3

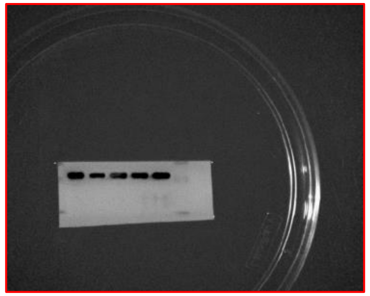
TPIS-1

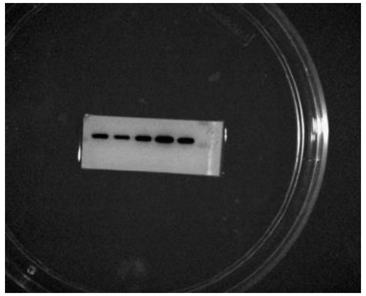
TPIS-2

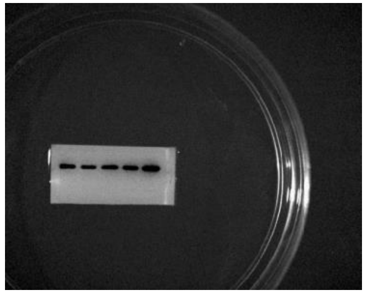
TPIS-3

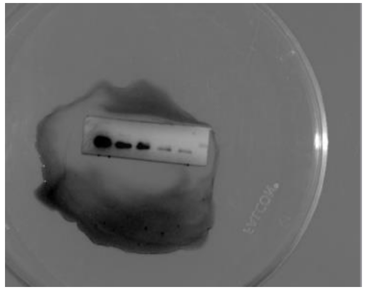
ALDOA-1

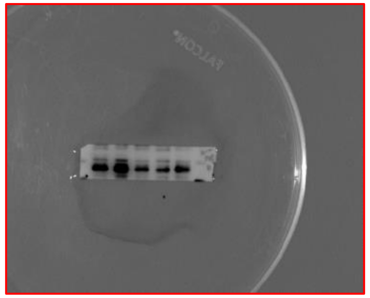
ALDOA-2

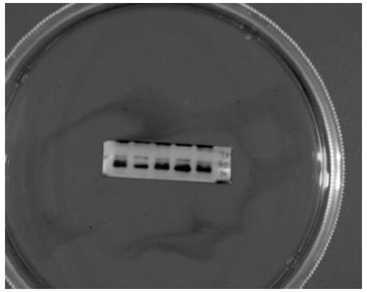
ALDOA-3

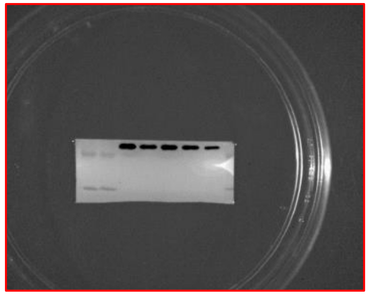
PGAM1-1

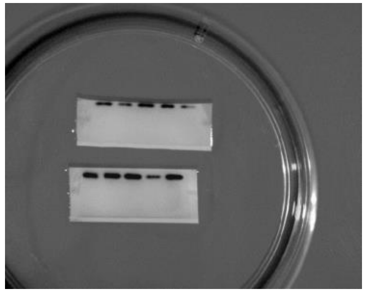
PGAM1-2/3

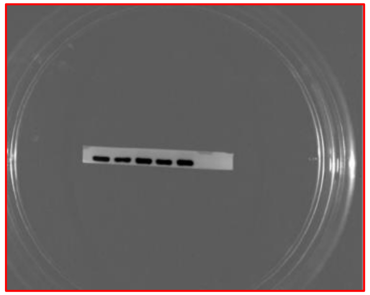
PGK1-1

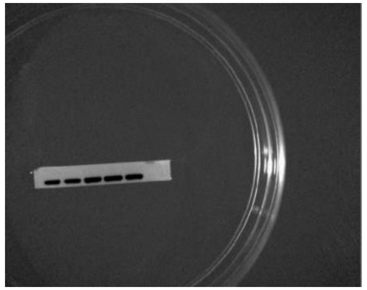
PGK1-2

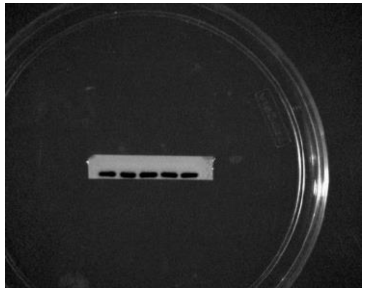
PGK1-3

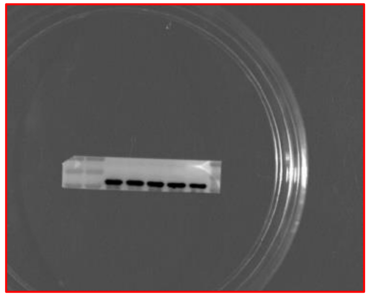
G6PI-1

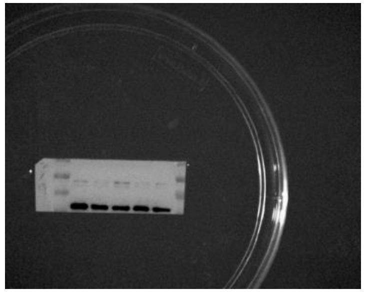
G6PI-2

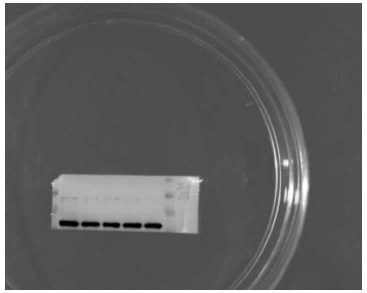
G6PI-3

Fig3C-Western blot analysis of glycolysis pathway proteins in whole cochlear tissue homogenates of noise-exposed and control C57BL/6 mice.

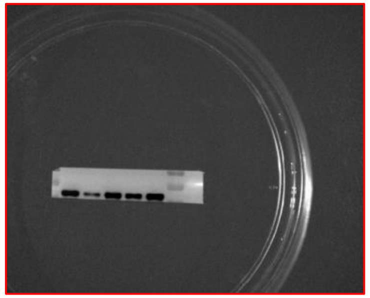

PKM-1

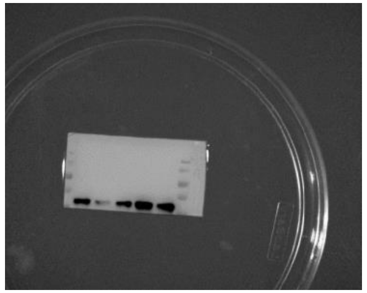

PKM-2

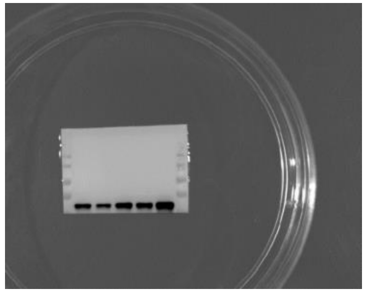

PKM-3

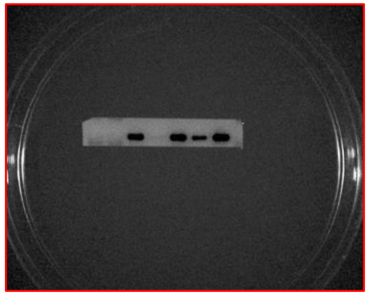

G3P-1

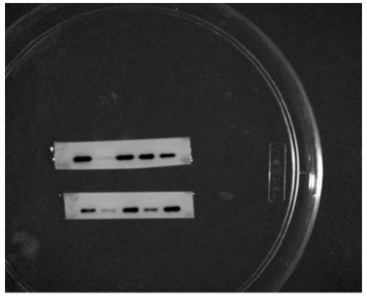

G3P-2/3

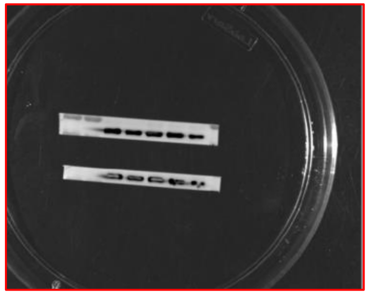

Actin-1

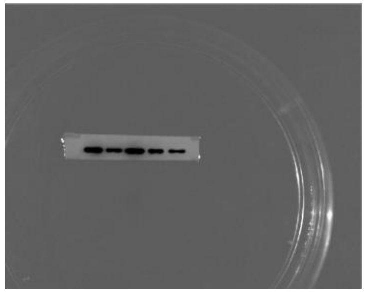

Actin-2

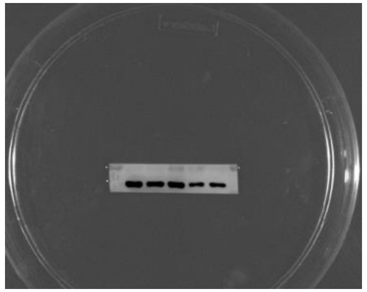

Actin-3

Fig4B-The protein expression of LDHA in SV-ECs with siLDHA knock down *in vitro*

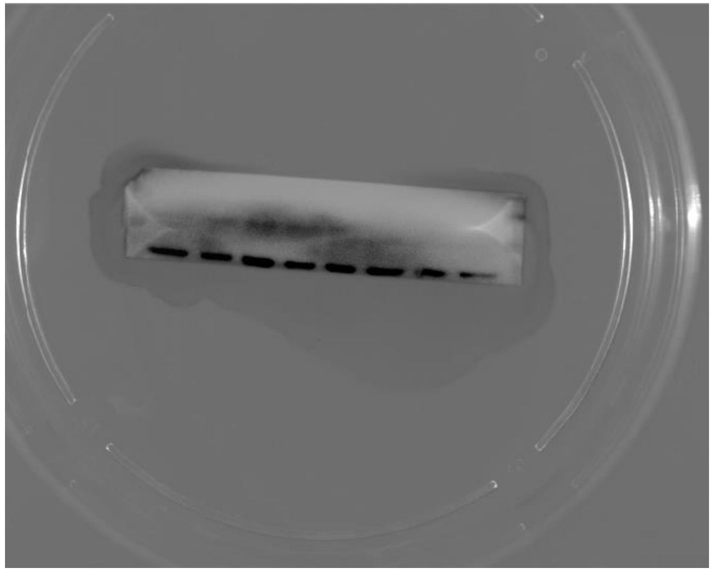

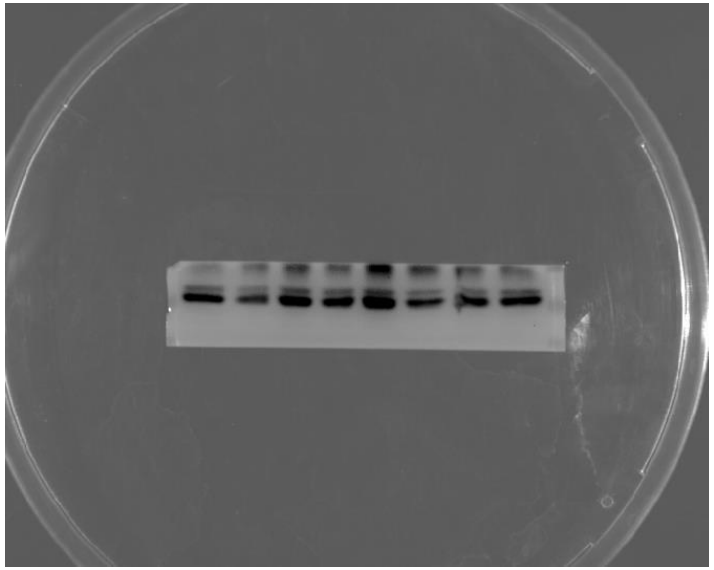

Actin

LDHA

FigS1-Western blots analysis of 3-NT in cochleae of C57BL6 mice exposed to noise and controls

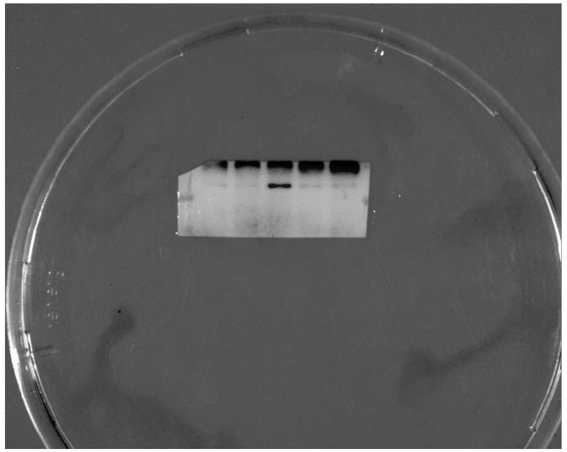

Actin-1

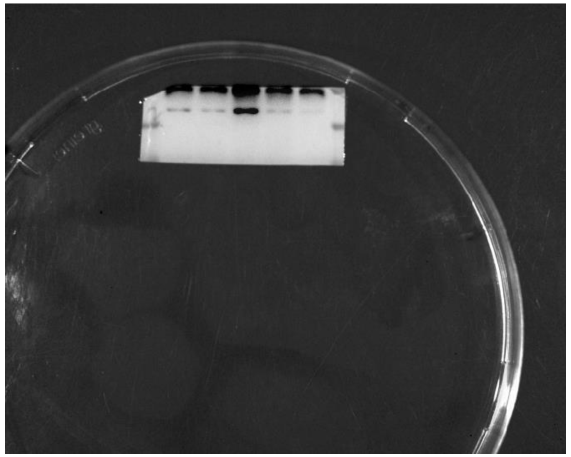

Actin-2

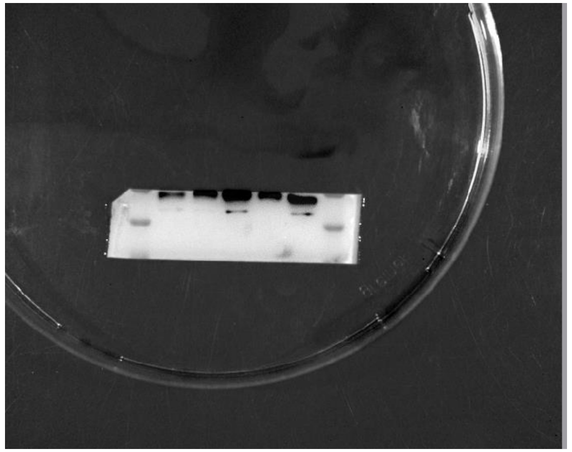

Actin-3

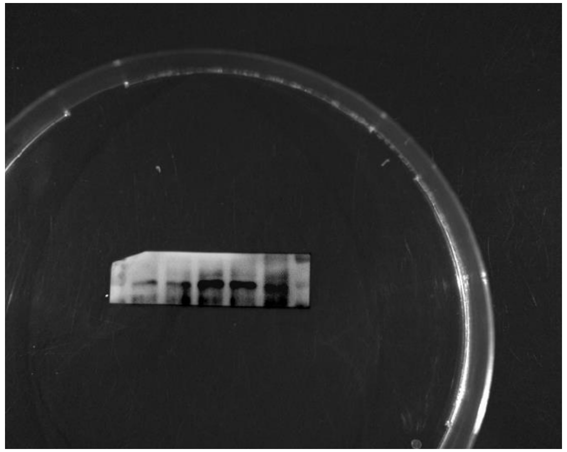

3NT-1

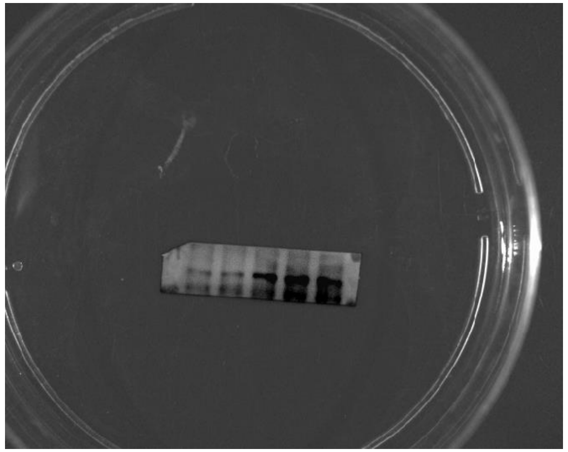

3NT-2

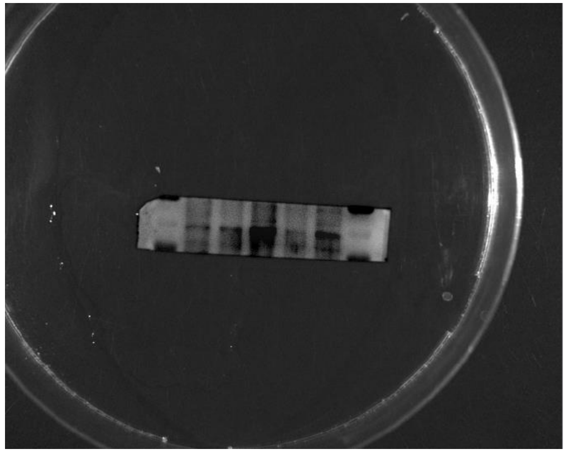

3NT-3

FigS2- Western blots analysis of glycolysis-related proteins in SV-ECs stimulation with 500μM H2O2 for 2 hours and subsequent cultured with SFM subsequently for 0, 6, 24 and 48 hours.

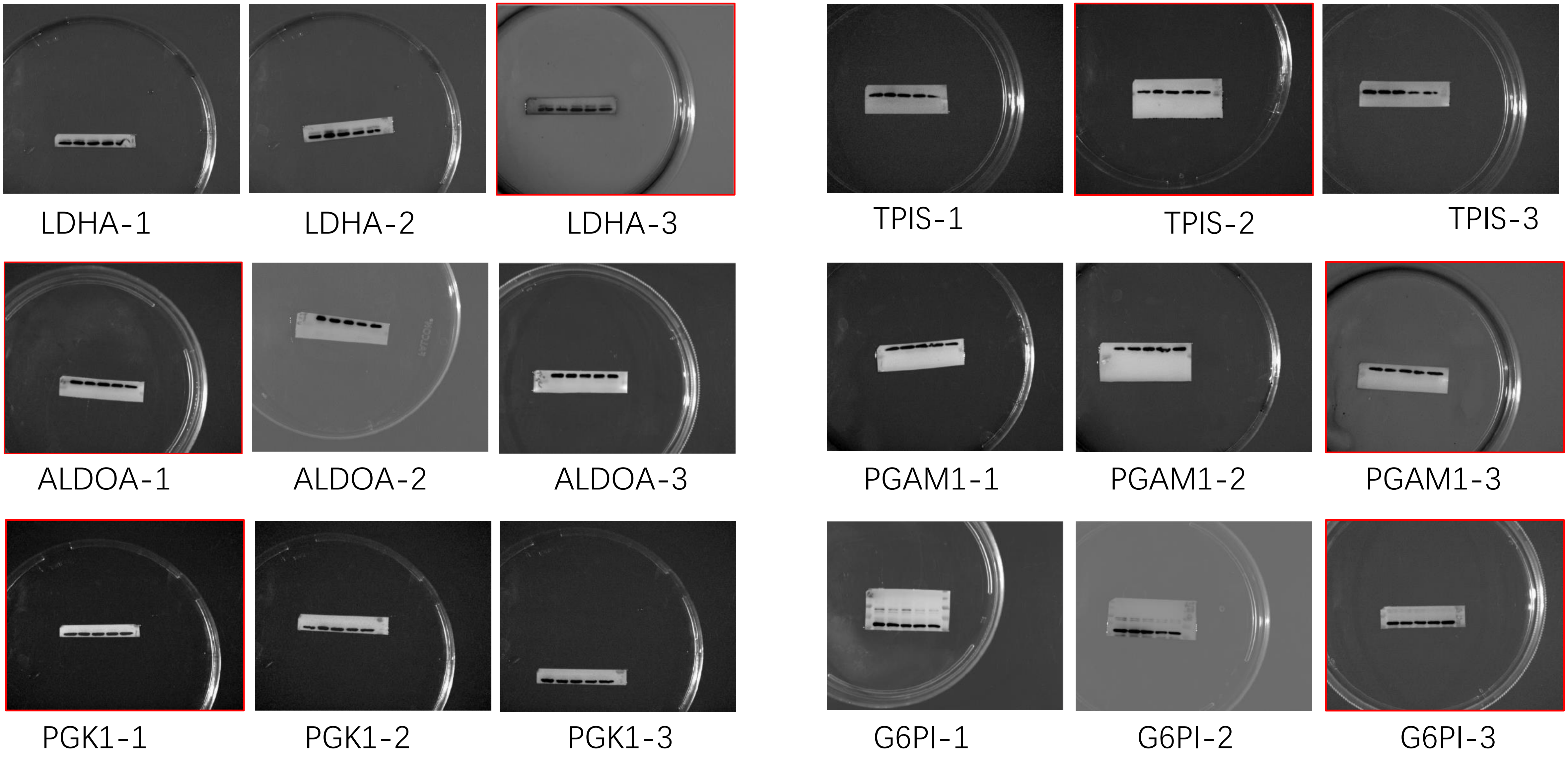

FigS2- Western blots analysis of glycolysis-related proteins in SV-ECs stimulation with 500μM H2O2 for 2 hours and subsequent cultured with SFM subsequently for 0, 6, 24 and 48 hours.

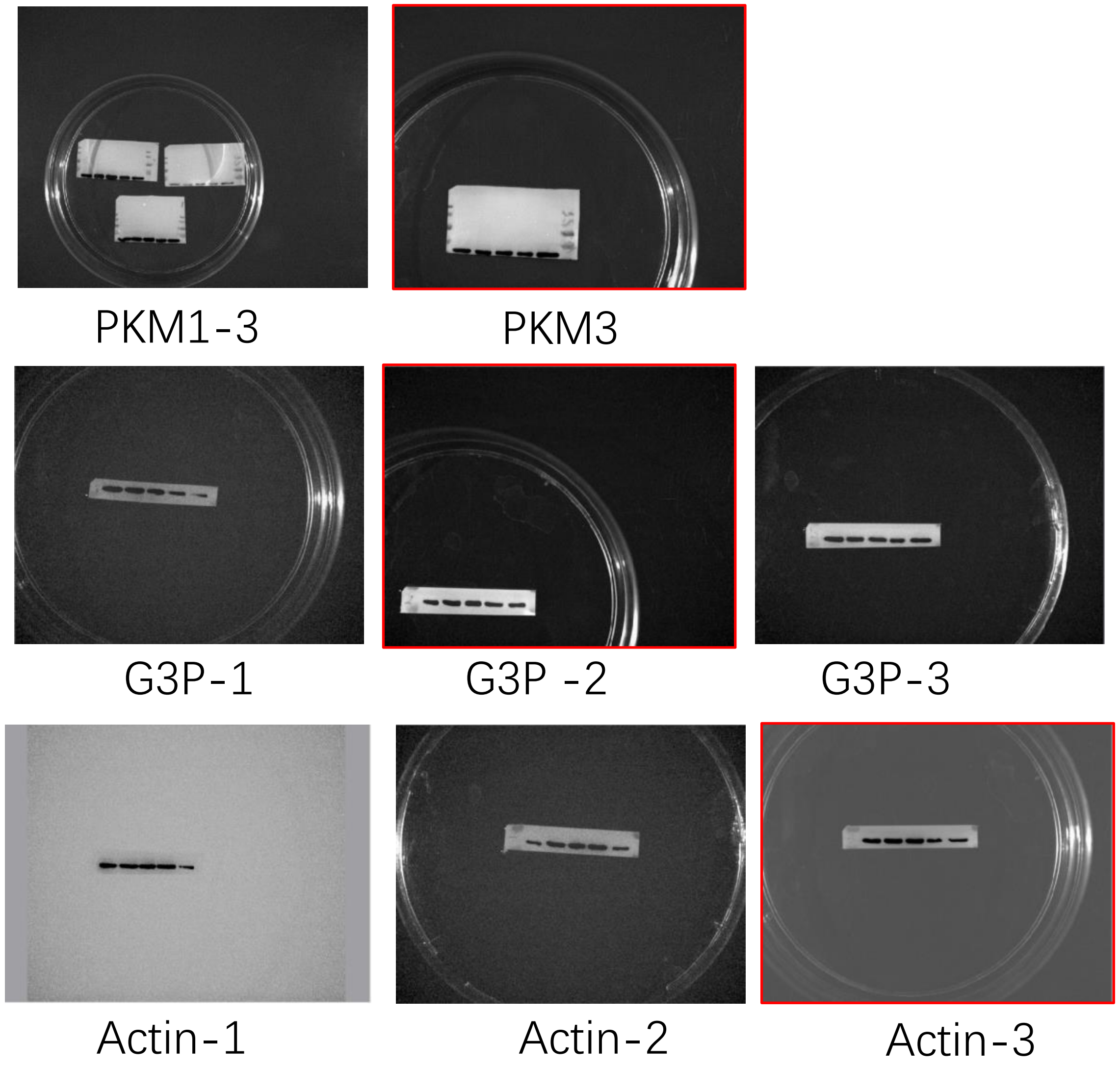

FigS4-The protein expression of of LDHA in SV with siLDHA knock down *in vivo*

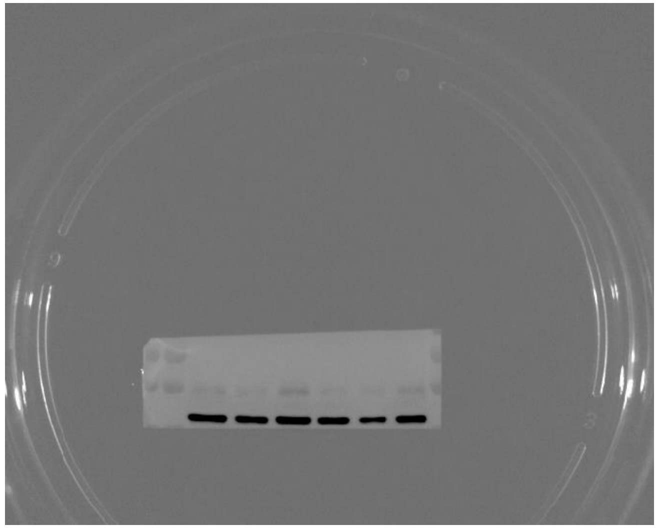

actin

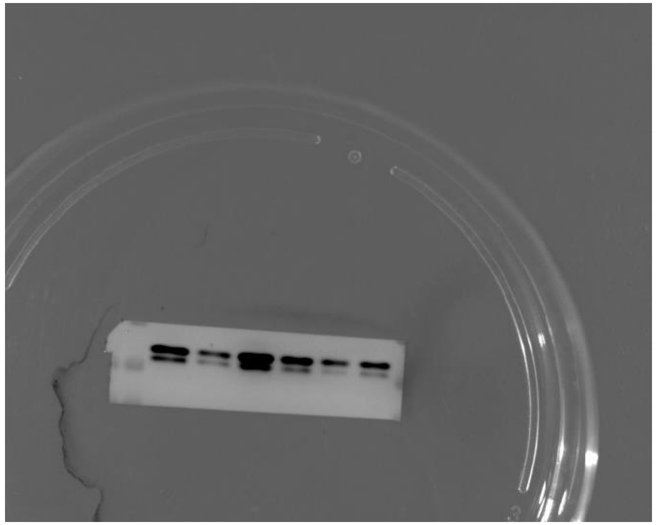

LDHA

FigS5-The protein expression of of CX3CL1 in SV-ECs with siCX3CL1 knock down *in vitro*

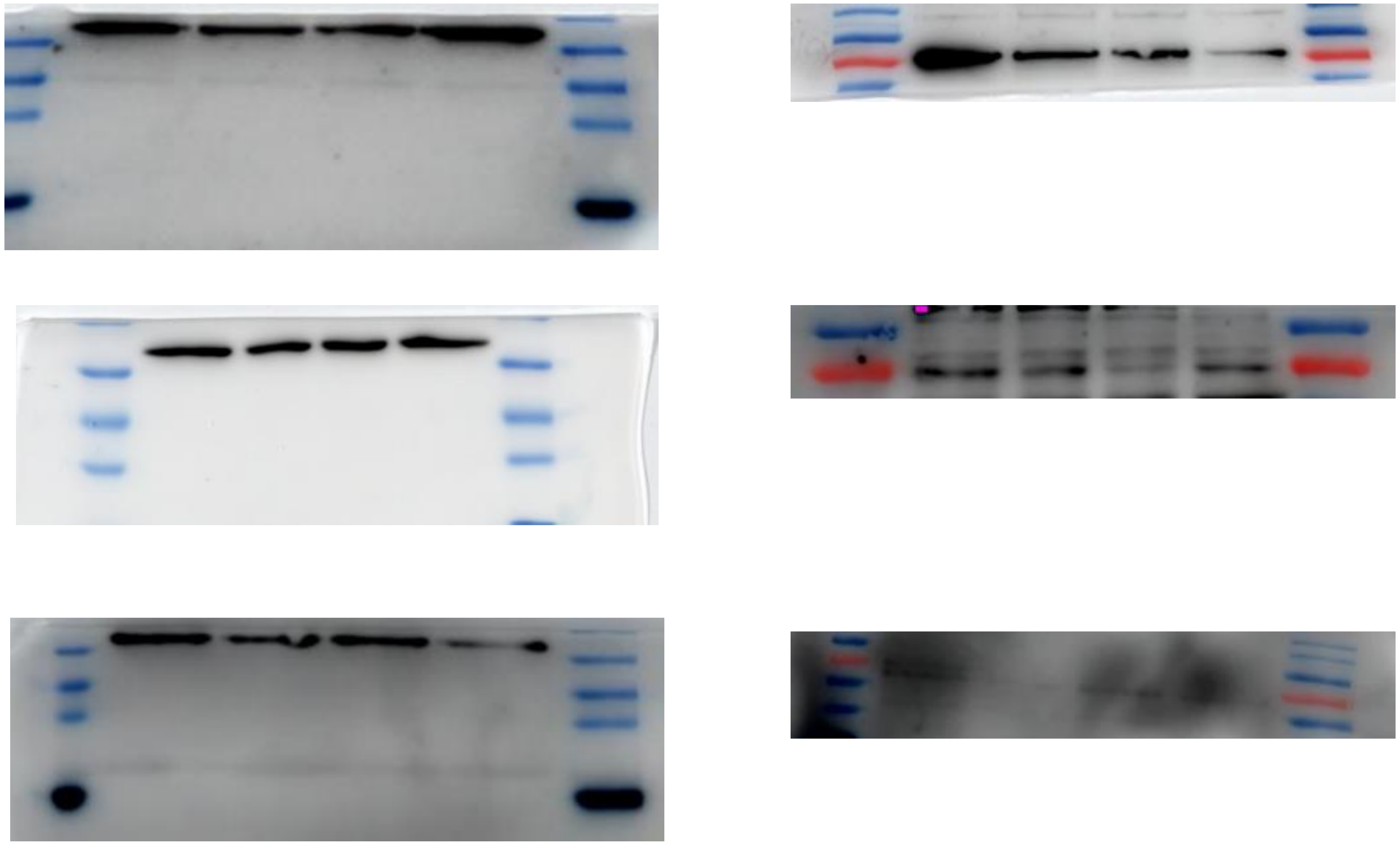

actin

CX3CL1
